# H_2_S-Prdx4 axis mitigates Golgi stress to bolster tumor-reactive T cell immunotherapeutic response

**DOI:** 10.1126/sciadv.adp1152

**Published:** 2024-11-15

**Authors:** Nathaniel Oberholtzer, Paramita Chakraborty, Mohamed Faisal Kassir, James Dressman, Satyajit Das, Stephanie Mills, Susana Comte-Walters, Monika Gooz, Seungho Choi, Rasesh Y. Parikh, Zacharia Hedley, Silvia Vaena, Reid DeMass, Gina Scurti, Martin Romeo, Vamsi K. Gangaraju, Stefano Berto, Elizabeth Hill, Lauren E. Ball, Anand S. Mehta, Eduardo N. Maldonado, Michael I. Nishimura, Besim Ogretmen, Shikhar Mehrotra

**Affiliations:** ^1^ Department of Surgery, Medical University of South Carolina, Charleston, SC 29425, USA.; ^2^ Department of Biochemistry and Molecular Biology, Medical University of South Carolina, Charleston, SC 29425, USA.; ^3^ Department of Cell and Molecular Pharmacology and Experimental Therapeutics, Medical University of South Carolina, Charleston, SC 29425, USA.; ^4^ Department of Drug Discovery and Biomedical Sciences, Medical University of South Carolina, Charleston, SC 29425, USA.; ^5^ Translational Science Laboratory, Hollings Cancer Center, Medical University of South Carolina, Charleston, SC 29425, USA.; ^6^ Department of Public Health, Medical University of South Carolina, Charleston, SC 29425, USA.; ^7^ Department of Surgery, Loyola University, Chicago, IL 60153, USA.; ^8^ Department of Neuroscience, Medical University of South Carolina, Charleston, SC 29425, USA.

## Abstract

The role of tumor microenvironment (TME)–associated inadequate protein modification and trafficking due to insufficiency in Golgi function, leading to Golgi stress, in the regulation of T cell function is largely unknown. Here, we show that disruption of Golgi architecture under TME stress, identified by the decreased expression of GM130, was reverted upon treatment with hydrogen sulfide (H_2_S) donor GYY4137 or overexpressing cystathionine β-synthase (CBS), an enzyme involved in the biosynthesis of endogenous H_2_S, which also promoted stemness, antioxidant capacity, and increased protein translation, mediated in part by endoplasmic reticulum–Golgi shuttling of Peroxiredoxin-4. In in vivo models of melanoma and lymphoma, antitumor T cells conditioned ex vivo with exogenous H_2_S or overexpressing CBS demonstrated superior tumor control upon adoptive transfer. Further, T cells with high Golgi content exhibited unique metabolic and glycation signatures with enhanced antitumor capacity. These data suggest that strategies to mitigate Golgi network stress or using Golgi^hi^ tumor-reactive T cells can improve tumor control upon adoptive transfer.

## Teaser

The Golgi apparatus can be targeted in antitumor T cells to enhance their immunotherapeutic capacity.

## INTRODUCTION

Adoptive transfer of tumor-reactive T cells has shown promising results in metastatic melanoma and advanced B cell malignancies (*1*, *2*). However, a quantitative or qualitative decrease of the transferred antitumor T cells in the tumor-bearing host typically results in tumor recurrence, leaving substantial room for improvement (*3*–*6*). Strategies to improve antitumor T cell function by altering mitochondrial bioenergetics (*7*, *8*) or its metabolites (*9*), mitigating endoplasmic reticulum (ER) stress (*10*), or inducing protective autophagy (*11*) are being widely tested. Multiple studies have identified oxidative and ER stress within the tumor microenvironment (TME) as major contributors of immune cell dysfunction and immune evasion (*12*–*16*). Similarly, the mammalian Golgi apparatus serves important roles in the transport, processing, and targeting of proteins and, when under stress, mounts a stress response where its unique structure can be fine-tuned to adapt different Golgi functions to specific cellular needs (*17*, *18*). While the synchronized activity of these cellular organelles is being increasingly recognized for maintaining quality control and ensuring cell survival and function (*19*), specifics of Golgi dynamics in the TME and the role of the Golgi stress response in shaping T cell function have thus far been understudied.

Similar to our recent study where carbon monoxide–mediated transient activation of the ER stress PERK pathway led to increased mitochondrial biogenesis and reprogramming of antitumor T cells to effectively treat established tumors upon adoptive T cell transfer (*11*), transient activation of Golgi stress mediated by monensin was shown to stimulate the reverse trans-sulfuration pathway via cystathionine γ-lyase (CSE) (the biosynthetic enzyme for cysteine and an important regulator of redox homeostasis) to mitigate the toxicity associated with cysteine deprivation in Huntington’s disease (*20*). Given that CSE-, cystathionine β-synthase (CBS)–, and 3-mercaptopyruvate sulfurtransferase (3-MST)–mediated secretion of hydrogen sulfide (H_2_S), an endogenous signaling gaseous transmitter that also mitigates Golgi stress (*17*), has been shown to regulate the immune response in mammals (*21*), we hypothesized that H_2_S may establish a reduced stress state in antitumor T cells and that increasing H_2_S could potentiate the antitumor T cell response by mitigating ER and Golgi stress.

Here, we show that T cell intrinsic H_2_S signaling supports overall protein translation and improves T cell effector function by reducing ER and Golgi stress. The levels of H_2_S or CBS also inversely correlated with exhaustion, and replenishing H_2_S exogenously during T cell activation or expansion led to an increase in the central memory (Tcm) phenotype by engaging the NAD^+^-Sirt1-Foxo1 axis. Proteomics analysis highlighted that increased abundance of free thiols in Peroxiredoxin-4 (Prdx4) was also in part responsible for the H_2_S-mediated Tcm phenotype. ER-localized Prdx4 was found to translocate to the Golgi under conditions of oxidative stress. Further, delineating T cells based on Golgi content highlighted that the T cell subsets with high Golgi content (Golgi^hi^ subset) exhibit long-term tumor control upon adoptive transfer. Further, human CD19 chimeric antigen receptor (CAR)–T cells overexpressing CBS or sorted for Golgi^hi^ significantly increased the survival of human lymphoma xenografted mice compared to mice treated with Golgi^lo^ CAR-T cells. Collectively, these results not only provide insight into the important role of endogenous H_2_S in regulating the T cell immune response but also highlight the Golgi network as a previously unidentified therapeutic target for enhancing the efficacy of immunotherapy for cancer.

## RESULTS

### H_2_S promotes generation of central memory antitumor T cells

**Fig. 1.**
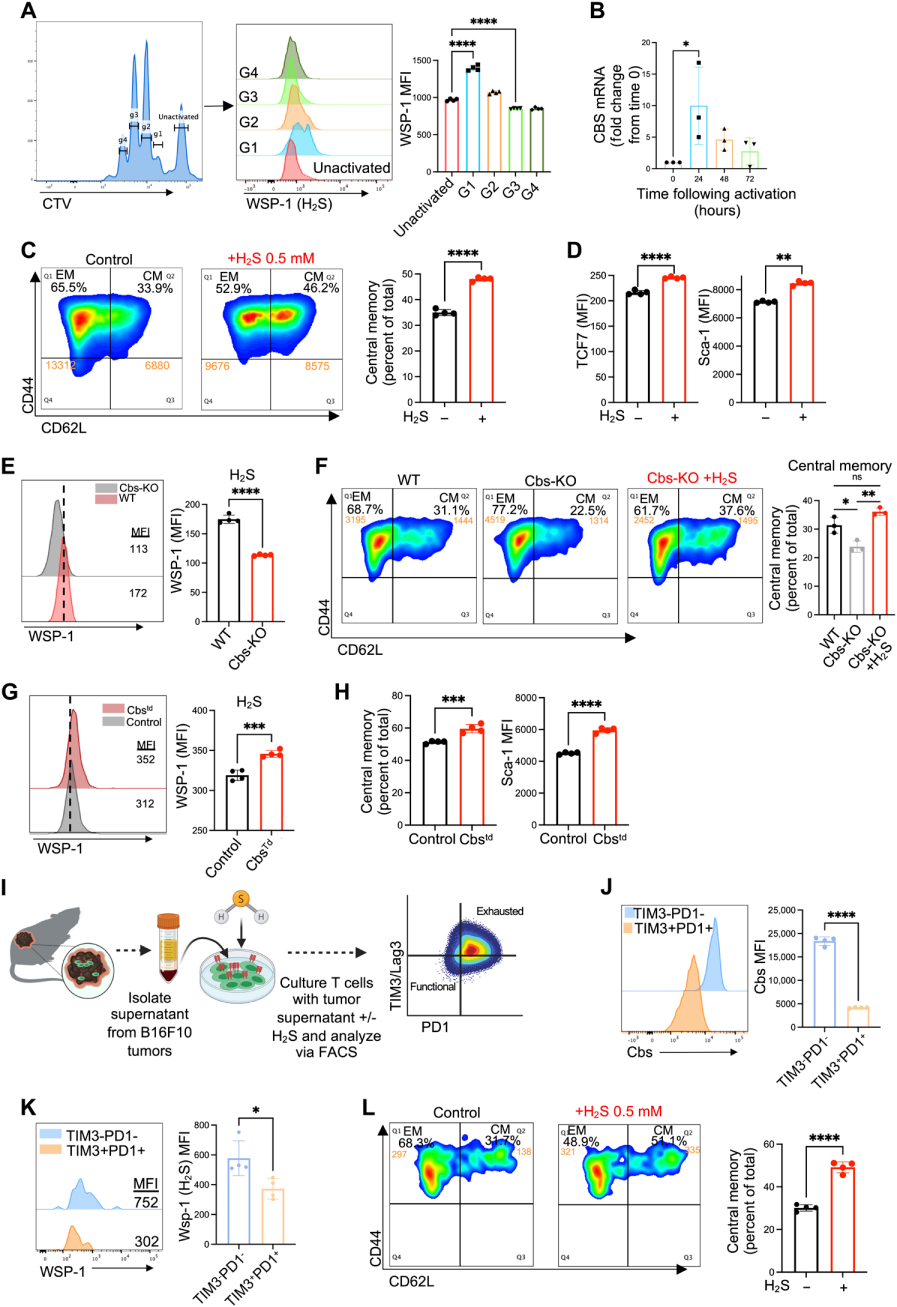
H_2_S promotes generation of central memory (Tcm) antitumor T cells. (**A**) Splenocytes from Pmel mice were stained with CTV dye and stimulated with gp100 antigen (1 μg/ml) + IL-2 (100 IU/ml) followed by staining with WSP-1 dye to quantify H_2_S production using FACS (*n* = 4 independent samples). (**B**) RT-PCR analysis performed on Pmel CD8^+^ T cells at various time points following activation to quantify mRNA levels of *CBS* (*n* = 4). (**C** and **D**) Pmel T cells treated with 0.5 mM GYY4137 for 3 days. (C) FACS analysis for expression of CD62L and CD44 (*n* = 4). (D) FACS analysis for expression of Tcf1/7 and Sca-1 (*n* = 4). (**E** and **F**) WT and Cbs-KO splenocytes activated with anti-CD3 and anti-CD28 and expanded with addition of H_2_S to the Cbs-KO cells. (E) Analysis of intracellular H_2_S by FACS using WSP-1 dye and (F) FACS analysis of relative percentage of CM to EM cells (*n* = 3). (**G** and **H**) Activated Pmel T cells transduced with lentiviral vector to overexpress CBS enzyme (Cbs^td^) and on day 7 analyzed for (G) intracellular H_2_S production using WSP-1 dye and (H) frequency of CM (CD62L^+^ CD44^+^) and Sca-1 expression (*n* = 4). (**I** to **L**) B16-F10 tumors implanted in C57BL/6 mice and resected on day 14 to culture in vitro. Supernatant from the cultured tumor cells was extracted and transferred to cultures containing Pmel T cells and gp100 antigen with or without 0.5 mM GYY4137. T cells cultured under the conditions of the exhaustion assay were analyzed for expression of (J) CBS by FACS and (K) intracellular H_2_S using WSP-1 dye and quantified by FACS (*n* = 4). (L) T cells from tumor supernatant exhaustion assay were also analyzed for the expression of CD62L and CD44 via FACS (*n* = 4). All data shown represent the mean ± SEM and were analyzed by two-sided Student’s *t* test or one-way analysis of variance (ANOVA). ns, *P* > 0.05; *P ≤ 0.05; **P ≤ 0.01; ***P ≤ 0.001; ****P ≤ 0.0001.

Given the limited knowledge on the role of H_2_S in primary T cells, we determined the kinetics of endogenous H_2_S production in antitumor T cells during T cell receptor (TCR)–mediated activation. Melanoma epitope gp100-reactive CD8^+^ T cells (from Pmel-transgenic mouse spleen) were stained with Cell Trace Violet (CTV) proliferation dye and activated in vitro with gp100 cognate antigen. After 3 days of activation, the T cells were stained with WSP-1 dye to quantify intracellular levels of H_2_S production in different generations. T cells within the first generation of proliferation (G1) displayed significantly increased levels of intracellular H_2_S compared to the naïve T cells (Fig. 1A). However, with progressive T cell proliferation (G2 to G4), intracellular H_2_S returned to basal levels. Corroborating these findings, mRNA samples collected from activated T cells before activation (time 0), and at 24, 48, and 72 hours demonstrated significant up-regulation of *Cbs* (one of the primary enzymes responsible for H_2_S production) 24 hours after activation, followed by a return to basal levels of expression by 72 hours (Fig. 1B).

We next determined whether restoring H_2_S levels in activated T cells would alter their phenotype. To achieve steady-state levels of H_2_S in the T cell culture media, we elected to use the water-soluble H_2_S donor GYY4137 that slowly releases sustained levels of H_2_S up to 7 days in culture (*22*). Given the cytotoxic nature of H_2_S at high concentrations, we selected 0.5 mM as the optimal dose of GYY4137 (fig. S1A) in all experiments where Pmel T cells were activated with cognate antigen gp100 for 7 days in presence of rIL2 (50 IU/ml) and gated on the CD8^+^ fraction for analysis (fig. S1B). We observed that T cells generated in the presence of H_2_S donor had a significant increase in the Tcm phenotype (as determined by CD62L^+^CD44^+^ coexpression) compared to those expanded with interleukin-2 (IL-2) alone (Fig. 1C), and this increase in Tcm phenotype was consistently maintained over 6 days (fig. S1C). Further analysis revealed that H_2_S treatment significantly enhanced the expression of Tcf7 and Sca1, markers related to the T cell stemness phenotype (Fig. 1D). A similar dose-dependent increase in the Tcm phenotype was observed when the fast-release H_2_S donor NaHS was used, added to the culture media daily (fig. S1D), supporting that H_2_S plays a role in generation of Tcm phenotype. A quantitative polymerase chain reaction (qPCR) analysis of the activated T cells that were FACS (fluorescence-activated cell sorting) sorted based on effector (CD62L^+^CD44^+^) and central memory (CD62L^+^CD44^+^) fraction showed increased expression of all three H_2_S-producing enzymes (*CBS*, *CSE*, and 3-*MST*) in the Tcm fraction, with *CBS* being most significantly up-regulated (fig. S1E). Treatment of T cells with IL-15 and IL-6, cytokines known to induce the Tcm phenotype in T cells (*23*) and play a key role in memory formation (*24*), respectively, resulted in significantly increased expression of both *CBS* and *CSE* (fig. S1F).

TCR-activated T cells from mice lacking CBS expression (Cbs-KO, fig. S1G) not only showed reduced H_2_S accumulation (Fig. 1E) but also exhibited reduced Tcm fraction when programmed in the presence of IL-15 (fig. S1H) and inferior ability to persistent following adoptive transfer compared to wild-type T cells (fig. S1I). However, adding H_2_S to the Cbs-KO T cells led to a restoration of the Tcm fraction (Fig. 1F). These data confirm that H_2_S levels play an important role in maintaining the Tcm phenotype. Overexpression of CBS (fig. S1J) in activated CD8^+^ T cells also resulted in a significantly enhanced H_2_S accumulation (Fig. 1G) and a concomitant increase in the Tcm population and Sca1 expression (Fig. 1H).

The solid TME is characterized as being highly immunosuppressive, leading to T cell exhaustion. Thus, we next used an in vitro model of TME-induced T cell exhaustion using supernatants collected from B16-F10 murine melanoma cells along with chronic antigen stimulation (*25*). Pmel CD8^+^ T cells were activated and cultured under optimal culture conditions, with tumor supernatant plus repeated stimulation with gp100, or with tumor supernatant plus repeated stimulation with gp100 plus H_2_S donor (Fig. 1I). This model was used to determine the impact of T cell exhaustion on CBS expression and H_2_S production by gating Pmel T cells on TIM3^+^PD1^+^ (terminally exhausted) and TIM3^−^PD1^−^ cells. We found that terminally exhausted T cells expressed significantly less CBS (Fig. 1J) with less H_2_S production (Fig. 1K). Addition of H_2_S to the culture not only enabled activated T cells to preserve the Tcm phenotype in the TME exhaustion assay (Fig. 1L) but also decreased expression of T cell exhaustion markers PD1, TIM3, Lag3, and CD38 (fig. S1K), suggesting a role for H_2_S in combating TME-induced T cell exhaustion. These data suggest that endogenous H_2_S levels play an important role in regulating T cell effector versus memory and exhaustion phenotypes.

### H_2_S supports T cell effector function and protein translation

**Fig. 2.**
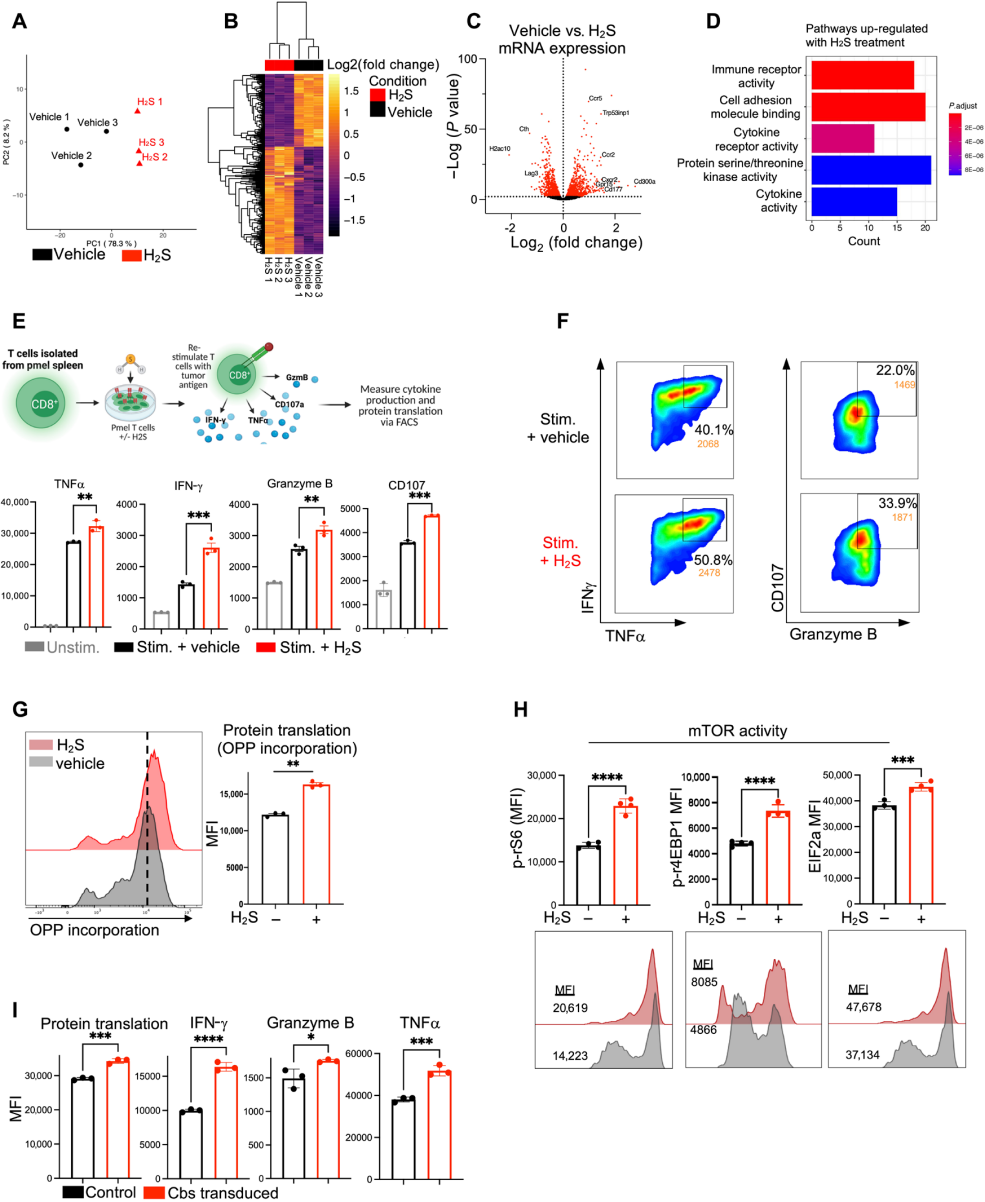
H_2_S supports T cell effector function and protein translation. (**A** to **D**) Melanoma epitope gp100 TCR reactive CD8^+^ T cells were activated for 3 days with gp100 and IL-2 with or without 0.5 mM GYY4137. On day 3, the cells were collected for RNA-seq analysis (*n* = 3 independent samples). (A) Principal components analysis (PCA) of control versus H2S-treated samples. (B) Heatmap displaying significantly up-regulated and down-regulated gene groups. (C) Volcano plot demonstrating significantly up-regulated and down-regulated genes using false discovery rate (FDR)–adjusted *P* value < 0.05. (D) Pathway enrichment analysis of significantly up-regulated genes. (**E**) Schematic of experimental design for cytokine and translation assays. (**F**) Pmel CD8^+^ T cells were activated for 3 days with gp100 and IL-2 with or without 0.5 mM GYY4137. On day 3, the cells were washed and restimulated with gp100 for 4 hours, followed by measurement of cytokine production via FACS. (**G**) Active protein translation was assessed using the Click-iT Plus OPP Alexa Fluor 647 Protein Synthesis Assay Kit (Thermo Fisher Scientific) (*n* = 3 independent samples) in Pmel T cells after restimulation (*n* = 3 independent samples). (**H**) Levels of EIF2a, phosphorylated 4EBP1, and phosphorylated rS6 evaluated via FACS in Pmel T cells after restimulation (*n* = 4 independent samples). (**I**) Pmel CD8^+^ T cells were activated for 2 days with gp100 and IL-2 followed by transduction with either control lentivirus or CBS-expressing lentivirus. On day 5, the cells were washed and re-stimulated with gp100 for 4 hours. Quantification of protein translation assessed using Click-iT Plus OPP Alexa Fluor 647 Protein Synthesis Assay Kit and quantification intracellular cytokines assessed using FACS (*n* = 3 independent samples). MFI represents mean fluorescence intensity. All data shown represent the mean ± SEM and were analyzed by two-sided Student’s *t* test or one-way ANOVA, unless otherwise specified. ns, *P* > 0.05; *P ≤ 0.05; **P ≤ 0.01; ***P ≤ 0.001; ****P ≤ 0.0001.

Next, we determined the effect of H_2_S treatment on the transcriptomic profile of antitumor T cells. Pmel CD8^+^ T cells were activated and expanded in the presence or absence of the H_2_S donor before RNA sequencing (RNA-seq). Principal components analysis (PCA) demonstrated a distinct transcriptomic profile for T cells treated with the H_2_S donor as compared to T cells activated under standard culture conditions (Fig. 2A). Of note, several of the most significantly up-regulated genes were genes involved in chemokine receptor activity and leukocyte migration, including *CCR2*, *CCR5*, *CXCR2*, *GRP15*, and *CD177* (Fig. 2, B and C). The major pathways significantly up-regulated in H_2_S-treated T cells included pathways related to immune receptor activity, T cell signaling, and cytokine activity (Fig. 2, C and D, and fig. S2A).

We next sought to determine the impact of H_2_S on T cell effector function. Pmel CD8^+^ T cells were activated and expanded with standard culture conditions or with the addition of the H_2_S donor and subsequently restimulated with gp100 cognate antigen (Fig. 2E). Pmel T cells treated with the H_2_S donor displayed enhanced secretion of effector cytokines tumor necrosis factor–α (TNFα) and interferon-γ (IFN-γ), as well as increased levels of cytolytic molecule granzyme B and CD107 expression (a marker of degranulation) (Fig. 2F) (*26*). Given the importance of protein translation in maintaining a robust antitumor response and the repression of T cell translation that occurs in the TME (*27*, *28*), we determined whether H_2_S influences protein translation. Notably, T cells treated with the H_2_S donor demonstrated significantly enhanced up-regulation of overall protein translation as measured by O-propargyl-puromycin (OPP) incorporation into the nascent polypeptide chain during translation upon restimulation with cognate antigen (Fig. 2G) (*29*). H_2_S-treated T cells also displayed increased total levels of EIF2α and phosphorylated levels of S6 ribosomal protein, essential factors regulating the rate-limiting step of protein synthesis, along with increased phosphorylation of 4EBP1 (one of the key negative regulators of protein synthesis which is inhibited by phosphorylation) (Fig. 2H). Similarly, Pmel CD8^+^ T cells transduced with CBS overexpressing vector displayed increased cytokine production and overall protein translation when reexposed to cognate tumor antigen (Fig. 2I), demonstrating that enhancing H_2_S signaling increases effector function in CD8^+^ T cells.

It has previously been shown that in CD4^+^ T cells, H_2_S promotes Tet-mediated Foxp3 demethylation to drive regulatory T cell (Treg differentiation) (*30*). However, recent studies have shown that reduced Tet2 expression results in the improved function of CD19 CAR-engineered T cells (*31*). We observed that in CD8^+^ T cells, H_2_S treatment results in a decrease of Tet activity (fig. S2B). These findings suggest distinct roles of H_2_S signaling in CD8^+^ T cell programming.

### H_2_S enhances antitumor efficacy of T cells in vivo

**Fig. 3.**
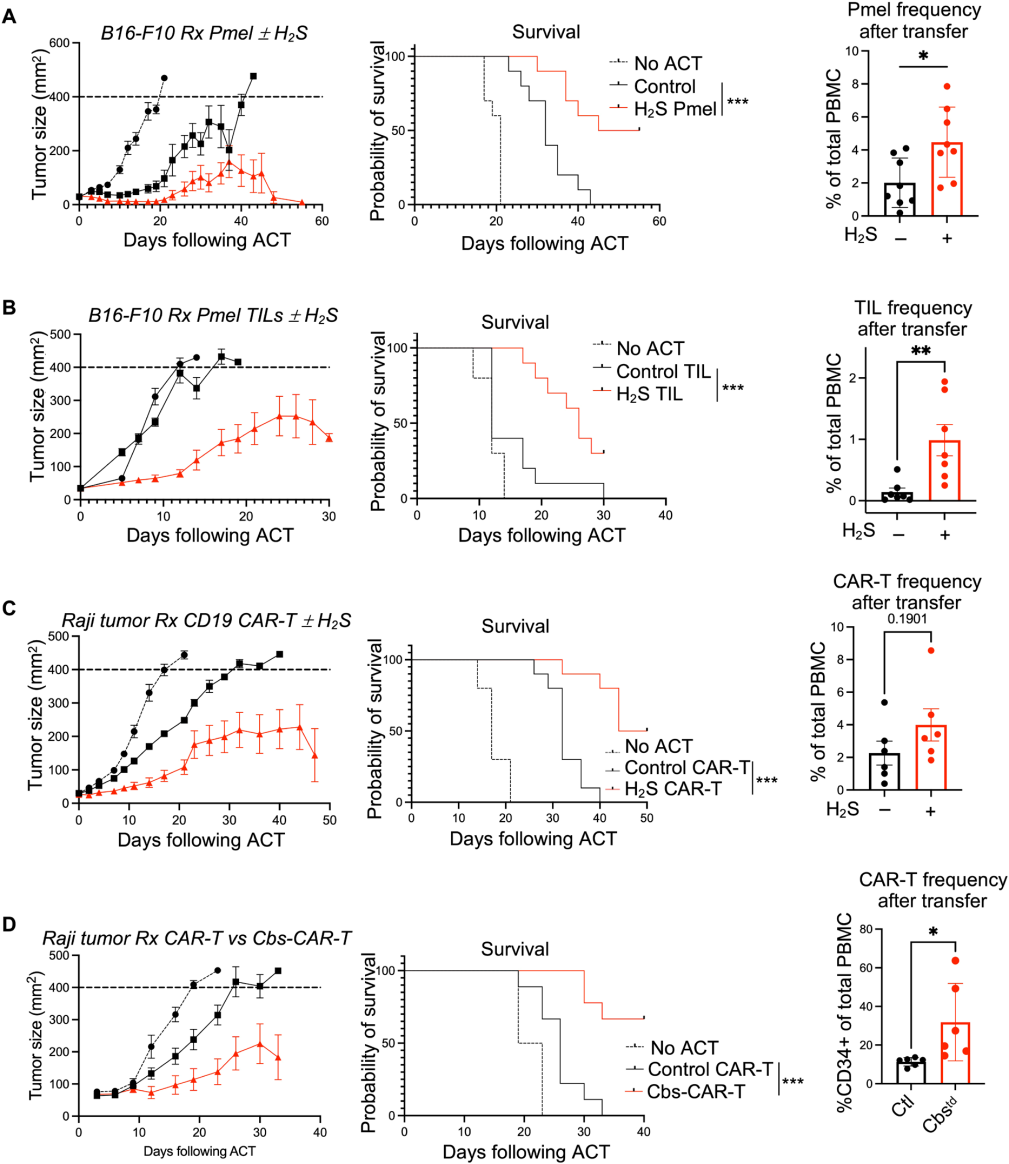
H_2_S enhances the antitumor efficacy of T cells in vivo. (**A** to **D**) Indicated cells were expanded ex vivo and transferred into mice bearing subcutaneously inoculated tumors. Tumor size was measured three times per week until end point size of 400 mm^2^ is reached. All tumor control experiments were repeated twice. (A) B16-F10 melanoma tumor-bearing C57BL/6 mice treated with melanoma epitope gp100 TCR reactive CD8^+^ T cells cultured with IL-2 alone or IL-2 + 0.5 mM GYY4137 (*n* = 10 mice per group). Frequency of Pmel T cells in peripheral blood at day 21 after transfer assessed by FACS. (B) B16F10 tumor-bearing C57BL/6 mice treated with TILs isolated from B16-F10 tumors grown subcutaneously on C57BL/6 mice and expanded with IL-2 alone or IL-2 + 0.5 mM GYY4137 (*n* = 10 mice per group). Frequency of transferred T cells in peripheral blood at day 10 after transfer assessed by FACS. (C) Raji tumor-bearing NSG mice treated with human CD19 CAR-T cells cultured with IL-2 alone or IL-2 + 0.5 mM GYY4137 (*n* = 10 mice per group). CAR-T cells were injected 3 days following Raji cell inoculation. Frequency of CD34^+^ transferred CAR-T cells in peripheral blood at day 21 after transfer assessed by FACS. (D) Human PBMCs were transduced with either CD34-CD19-CAR-T construct or Cbs-CD34-CD19-CAR-T construct. Transduced CAR-T cells were sorted on CD34^+^ cells and adoptively transferred into CD19^+^ Raji tumor-bearing NSG mice 6 days after Raji cell inoculation (*n* = 10 mice per group). Frequency of CD34^+^ transferred CAR-T cells in peripheral blood at day 21 after transfer assessed by FACS. For all survival outcomes, Kaplan-Meier curves were used to display the results. Median survival time and corresponding 95% confidence interval were calculated for each experimental condition. A log-rank test was used to compare the outcomes across experimental conditions. ns, *P* > 0.05; *P ≤ 0.05; **P ≤ 0.01; ***P ≤ 0.001.

Given the Tcm phenotype, reduced exhaustion, and enhanced effector function of T cells treated with H_2_S in vitro, we next assessed the utility of H_2_S-based strategies in tumor control. First, we used Pmel T cells expanded with or without the H_2_S donor that were subsequently transferred to B16-F10 murine melanoma-bearing immunocompetent C57BL/6 mice (fig. S3A, schematic). The H_2_S-treated Pmel T cells displayed a superior ability to control tumor growth and prolong overall survival compared to control Pmel T cells (Fig. 3A), which also correlated with increased persistence (right panel). Enhanced CD62L^+^CD44^+^ Tcm phenotype and Sca1 expression was also noted in the group that received H_2_S-pretreated T cells (fig. S3B). Next, to determine whether H_2_S treatment would reprogram tumor-infiltrating lymphocytes (TILs) and render them more effective, we obtained TILs from B16-F10 tumor engrafted in Pmel mice and then expanded them with or without the H_2_S donor (fig. S3C, schematic). Following adoptive transfer into tumor-bearing mice, we observed that mice receiving H_2_S-treated TILs exhibited significant reduction in tumor growth and extended overall survival (Fig. 3B), which also correlated with its enhanced persistence.

Given the demonstrated efficacy of using H_2_S to enhance adoptive cell therapy (ACT) protocols in murine models of melanoma, we determined whether this strategy would be equally efficacious in controlling human tumors. Thus, human B cell lymphoma Raji cells engrafted in NSG mice were treated with human CD19 CAR-transduced T cells that were generated in the presence or absence of H_2_S (fig. S3D, schematic). As expected, mice receiving CD19 CAR-T cells expanded with H_2_S had a significant reduction in tumor growth and an increase in overall survival (Fig. 3C), as well as increased persistence (right panel). To further support the translational applicability of these findings, we designed a CAR-T construct to incorporate *Cbs*. These CD19-Cbs–engineered CAR-T cells (Cbs^td^ CAR-T cells) also exhibited better tumor control as compared to the CD19 CAR-engineered T cells (Fig. 3D) with increased persistence (right panel). This suggests that antitumor T cells with increased H_2_S signaling exhibit better persistence in vivo and CAR-T cells engineered to express CBS can potentiate tumor control.

### H_2_S alters metabolic profile and enhances the mitochondrial function of T cells

**Fig. 4.**
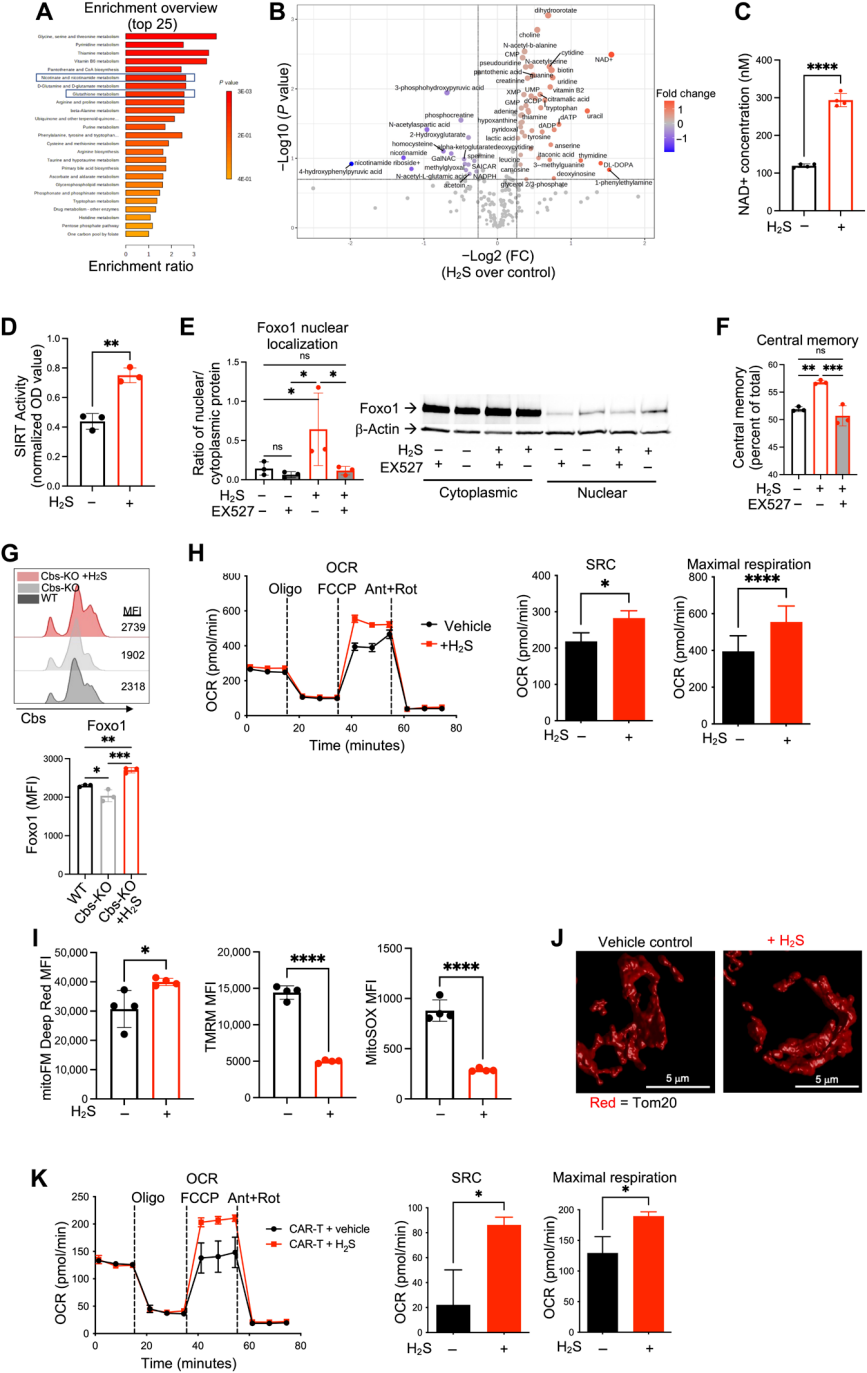
H_2_S alters metabolic profile and enhances the mitochondrial function of T cells. (**A** to **C**) Pmel T cells activated for 3 days with gp100 and IL-2 with or without 0.5 mM GYY4137 and collected for comprehensive metabolomics analysis. (A) Pathway enrichment analysis based on significantly different metabolites. (B) Volcano plot showing significantly up-regulated and down-regulated metabolites based on –log10(*P*) > 1. (C) NAD^+^ and nicotinamide quantification from metabolomics dataset. (**D**) SIRT1 deacetylase activity in activated Pmel T cells (means of normalized OD values). (**E** and **F**) Pmel T cells were activated with gp100 for 3 days in the presence of vehicle, GYY4137 (0.5 mM), or GYY4137 + sirt1 inhibitor EX527 (10 μM). (E) Nuclear and cytoplasmic protein fractions were isolated and Western blot analysis was performed to determine the relative expression of nuclear and cytoplasmic Foxo1 (*n* = 3). (F) Expression of CD62L and CD44 analyzed by FACS (*n* = 3). (**G**) Splenocytes from wild-type and Cbs-knockout mice +/- GYY4137 activated with anti-CD3 and anti-CD28 antibodies followed by intranuclear staining for Foxo1. (**H**) Pmel T cells activated and expanded for metabolic flux analysis. Quantification of oxygen consumption rate (OCR), spare respiratory capacity (SRC), maximal respiration, and basal respiration (*n* = 10 experimental replicates representative of three separate experiments). (**I**) Pmel T cells activated and expanded to day 5 for characterization of mitochondria using FACS, quantified by MFI values for mitoFM Deep Red, TMRM, and mitoSOX dyes (*n* = 4). (**J**) Representative confocal microscopy images of mitochondria from control or H_2_S-treated T cells. (**K**) Human CD19 CAR-T cells expanded for 3 days with IL-2 with or without 0.5 mM GYY4137 and collected for metabolic flux analysis. Shown is the quantification of mean OCR, SRC, maximal respiration, and basal respiration. Data shown represent the mean ± SEM and were analyzed by two-sided Student’s *t* test or one-way ANOVA. ns, *P* > 0.05; *P ≤ 0.05; **P ≤ 0.01; ***P ≤ 0.001; ****P ≤ 0.0001.

Next, we sought to characterize the metabolic status of antitumor T cells treated with exogenous H_2_S. Comprehensive metabolomics analysis revealed that Pmel CD8^+^ T cells treated with the H_2_S donor possessed a distinct profile of metabolites relating to enrichment of several metabolic pathways (Fig. 4A and fig. S4A). Of note, several of the top pathways affected by H_2_S treatment involved metabolic pathways that are known to be critical for antitumor immunity, including serine, vitamin B6, and nicotinamide metabolism (Fig. 4A) (*9*, *32*). Specifically, the pathway of nicotinate and nicotinamide metabolism was enriched, with increased levels of NAD^+^ and decreased levels of nicotinamide in H_2_S-treated T cells (Fig. 4, B and C). This corresponded to increased expression of NAD(P)H quinone oxidoreductase 1 (NQO1) identified in the RNA-seq analysis, an antioxidative enzyme that modulates the differentiation of T_H_17 cells by regulating reactive oxygen species (ROS) levels (*33*). These data imply that H_2_S renders increased antioxidant capacity to T cells, which may contribute to increase persistence and Tcm phenotype (as reported earlier) (*2*, *34*).

Of note, an increase in levels of NAD^+^ has been linked to the enhanced antitumor efficacy of CD8^+^ T cells through its key role as a substrate for Sirt1 (*9*). We found that the H_2_S-treated T cells were also characterized by increased Sirt1 activity (Fig. 4D). Previous studies have shown that the transcription factor Foxo1 regulates the Tcm phenotype (*35*) and that NAD^+^-dependent Sirt1 is responsible for its deacetylation and activity (*9*). Recent studies have also shown that Foxo1 overexpression promotes a stem-like phenotype in CAR-T cells derived from either healthy human donors or patients, which correlates with improved mitochondrial fitness, persistence, and therapeutic efficacy in vivo (*36*, *37*). Thus, we probed whether H_2_S treatment increased nuclear translocation of Foxo1 via enhanced Sirt1 activity in T cells. We observed that H_2_S treatment led to increased nuclear localization of Foxo1 and that Sirt1 inhibitor Ex527 treatment neutralized H_2_S-mediated nuclear localization of Foxo1 (Fig. 4E) and Tcm phenotype (Fig. 4F). Expression of Foxo1 was reduced in Cbs-KO T cells and was restored upon H_2_S treatment (Fig. 4G). These data indicate that the H_2_S-mediated increase in Tcm phenotype may be dependent on Sirt1-mediated enhancement of nuclear localization of Foxo1.

Next, given the distinct metabolite profile observed in H_2_S-treated T cells, we analyzed the mitochondrial fitness of the T cells generated in the presence of H_2_S. We found that Pmel T cells activated in the presence of the H_2_S donor did not alter basal respiration but resulted in a significant increase in both maximal respiration and spare respiratory capacity (SRC) (Fig. 4H). Further, the Pmel T cells treated with the H_2_S donor had increased overall mitochondrial mass as measured by mitoFM staining, decreased mitochondrial membrane potential as measured by tetramethylrhodamine methyl ester (TMRM) staining, and decreased mitochondrial ROS as measured by mitoSOX staining (Fig. 4I). Notable differences in mitochondrial organization and morphology were also observed with H_2_S treatment, including more extended and dispersed mitochondria in the control group compared to more compact and continuous mitochondria in the H_2_S-treated cells (Fig. 4J). Similarly, the human CD19 CAR-T cells treated with the H_2_S donor displayed a significant increase in maximal respiration and SRC (Fig. 4K). Given the previously demonstrated importance of low mitochondrial membrane potential and low mitochondrial ROS in metabolically fit antitumor T cells with enhanced antioxidant capacity and stemness (*8*, *34*), these findings suggest an overall increase in mitochondrial fitness in the presence of H_2_S.

### H_2_S reduces oxidative stress and Golgi-ER network stress in antitumor T cells

**Fig. 5.**
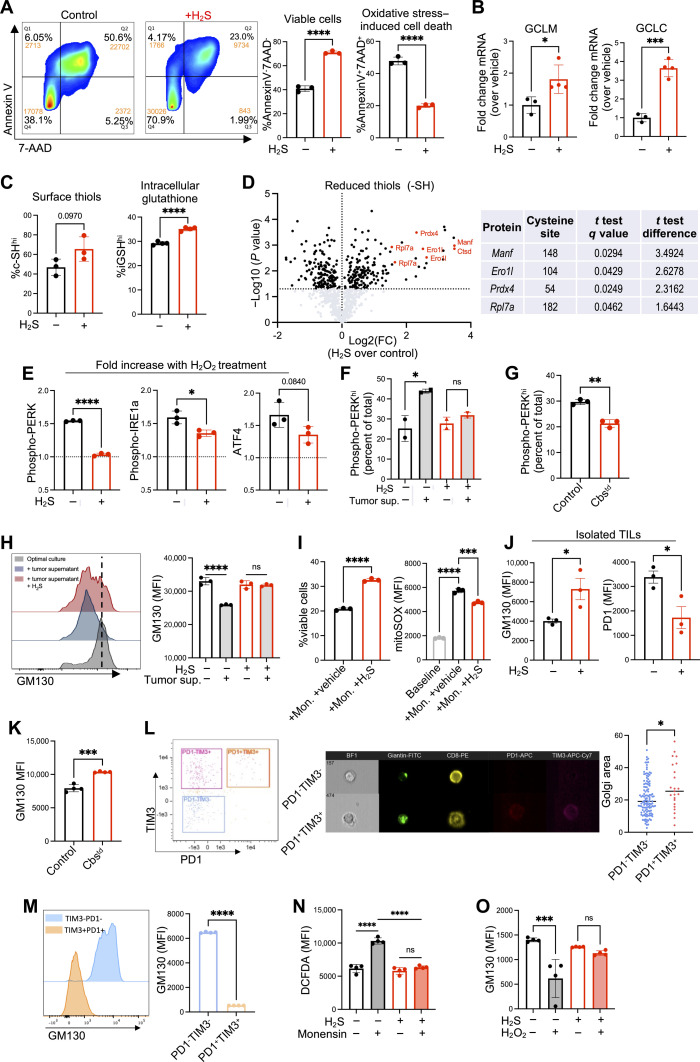
H_2_S reduces oxidative stress and Golgi-ER network stress in antitumor T cells. (**A** to **F**) Pmel T cells activated for 3 days with or without 0.5 mM GYY4137. (A) Cells treated for 12 hours with H_2_O_2_ (50 mM). FACS analysis quantifying 7-AAD^+^annexin V^+^ and 7-AAD^−^annexinV^−^ populations (*n* = 3). (B) mRNA levels of *GCLM* and *GCLC*. (C) Measurements of surface thiols by Alexa Fluor 488 C_5_ maleimide (*n* = 3) and intracellular glutathione by ThiolTracker Violet (*n* = 4). (D) Proteomics analysis quantifying thiol modifications on cysteine residues with statistically significant results based on one-sample *t* test with FDR < 0.5 with a median of three normalized ratios using log2(fold change treated versus control) (*n* = 3). (E) Fold increase in MFI for phosphorylated-PERK, phosphorylated-IRE1a, and ATF4 after 12-hour incubation with 50 mM H_2_O_2_ (*n* = 3). (F) FACS quantification of cells expressing high phosphorylated-PERK levels after exhaustion assay (*n* = 2). (**G**) Percentage of cells expressing high phosphorylated-PERK in Pmel cells overexpressing CBS compared to Pmel cells transduced with control lentivirus (*n* = 3). (**H**) Fold change in GM130 MFI in Pmel cells using exhaustion assay (*n* = 3). (**I**) Cell viability and mitochondrial ROS following monensin treatment (1 mM). (**J**) MFI values for GM130 and PD1 in TILs isolated from B16-F10 tumors and expanded +/- GYY4137 (*n* = 3). (**K**) GM130 MFI values in Pmel T cells overexpressing CBS compared to control cells after transfer into tumor-bearing mice and TIL isolation after 7 days (*n* = 4). (**L**) Pmel T cells cultured with B16-F10 supernatant and gp100, followed by FACS sorting for PD1^+^TIM3^+^ and PD1^−^TIM3^−^ cells. Sorted cells stained with anti-Giantin antibody and analyzed for Golgi area via ImageStream. (**M**) FACS analysis of GM130 expression in PD1^+^TIM3^+^ and PD1^−^TIM3^−^ populations. (**N**) Pmel cells treated with 1 mM monensin and analyzed for intracellular ROS (DCFDA). (**O**) FACS analysis of GM130 expression in Pmel cells treated with 50 mM H_2_O_2_ (6 hours). Data represent mean ± SEM. Analyzed by two-sided Student’s *t* test or one-way ANOVA. ns, *P* > 0.05; *P ≤ 0.05; **P ≤ 0.01; ***P ≤ 0.001; ****P ≤ 0.0001.

Reducing T cell intrinsic ROS generation has been shown to alleviate T cell exhaustion and improve the efficacy of T cell immunotherapy. Given that H_2_S has been identified to up-regulate cellular antioxidant defense mechanisms (*38*–*40*), we next sought to determine its ability to combat oxidative stress in antitumor T cells. Using hydrogen peroxide (H_2_O_2_) to induce oxidative stress in vitro, Pmel CD8^+^ T cells treated with the H_2_S donor exhibited a significant decrease in apoptotic cell death (Fig. 5A). H_2_S treatment also resulted in increased expression of glutamate cysteine ligase (GCL) holoenzyme catalytic subunit (GCLC) and modifier subunit (GCLM) (Fig. 5B), which catalyzes the rate-limiting step in the formation of the cellular antioxidant glutathione (GSH) to maintain cellular GSH homeostasis. In line with this observation, we noted that H_2_S-treated Pmel T cells and CD19 CAR-T cells overexpressing CBS exhibited an increase in overall cell-surface thiol (-SH) expression and intracellular glutathione (iGSH) (Fig. 5C and fig. S5A), correlating to their increased resistance to oxidative stress–induced cell death. Given the observed increase in total surface thiols, we next used a proteomic approach to identify proteins with cysteine thiols sensitive to H_2_S treatment (table S1). Our proteomics data revealed several key proteins involved in regulating ER stress that display enhanced abundance or an increase in the extent of free thiols in response to H_2_S treatment (table S2), including the key ER localized proteins Prdx4, ER oxidoreductin 1 (Ero1), mesencephalic astrocyte-derived neurotrophic factor 1 (MANF1), and the 60S ribosomal protein L7a (Rpl7a) (Fig. 5D).

While Prdx4 supports redox homeostasis by metabolizing H_2_O_2_ in the ER, its loss leads to oxidative stress and toxicity (*41*). Ero1 is an oxidoreductase enzyme that catalyzes the formation and isomerization of protein disulfide bonds in the ER, generating H_2_O_2_ in the process (*42*). Given the ER-Golgi intricate association and that Golgi stress response has been shown to reprogram cysteine metabolism (*20*), we hypothesized that H_2_S levels regulate oxidative stress within the ER and Golgi network. We observed that markers of ER stress, including phospho-PERK, phospho-IRE1a, and ATF4, were increased when control Pmel T cells were exposed to oxidative stress; however, this increase was mitigated in H_2_S-treated T cells (Fig. 5E). The Cbs-KO T cells also exhibited increased ER stress, measured by increased pPERK, which was reduced in the presence of H_2_S (fig. S5B). We also noted a significant increase in ER stress in T cells exposed to the in vitro TME exhaustion model, which was mitigated with H_2_S treatment (Fig. 5F). Further, overexpression of CBS resulted in a decrease in percentage of T cells experiencing high levels of ER stress (phospho-PERK^hi^) (Fig. 5G).

Golgi stress has recently been identified as an important mediator of redox imbalance in human cells (*43*), and H_2_S signaling has also been shown to be protective against Golgi stress (*17*). To track Golgi stress, we chose to determine the expression of GM130, a Golgi tethered protein that has been shown to control Golgi morphology in response to changes in cellular conditions (*44*). Previous studies have shown that Purkinje neurons in mice that lack the GM130 exhibit Golgi fragmentation and decreased secretory trafficking, leading to ataxia and cell death (*45*). Thus, we hypothesize that strategies that maintain Golgi homeostasis and avoid Golgi disruption in T cells would also result in increased persistence and preserve effector functions in the TME. Induction of T cell exhaustion using the TME exhaustion model resulted in Golgi stress, as measured by a significant decrease in GM130 expression (Fig. 5H). This decrease in GM130 expression was mitigated in the presence of the H_2_S donor (Fig. 5H). To further determine the impact of Golgi stress on T cells, we used a known Golgi stressor, monensin, to induce Golgi stress in Pmel T cells in the presence or absence of H_2_S. Monensin treatment induced cell death and cellular accumulation of mitochondrial ROS, which was partially mitigated with H_2_S treatment (Fig. 5I). TILs isolated from B16-F10 tumors and expanded under normal control conditions (without H_2_S) or with H_2_S revealed significantly higher GM130 expression and lower PD1 expression in the H_2_S-treated group (Fig. 5J). These results demonstrate that Golgi stress is induced in the TME and that H_2_S can be used to reprogram TILs to reduce Golgi stress, correlating to a decrease in T cell exhaustion. Similarly, Pmel T cells transduced with CBS and adoptively transferred into tumor-bearing mice maintained significantly higher GM130 expression when isolated from the tumors (Fig. 5K). Monensin did not induce ER stress, nor did thapsigargin induced Golgi stress (fig. S5C), highlighting the exclusivity of organelle stress and need for mitigating them is important to obtain a “stress-free” T cell.

Golgi dispersion under conditions of stress has been identified as a key feature of Golgi dysfunction (*44*). Thus, we induced Golgi stress with monensin and characterized Golgi dispersion with ImageStream analysis, showing that Golgi dispersion significantly increased when T cells were treated with monensin (fig. S5D). Next, we induced T cell exhaustion using the previously described in vitro TME model and characterized Golgi dispersion in terminally exhausted T cells (PD1^+^TIM3^+^) compared to healthy T cells (PD1^−^TIM3^−^). Like monensin treatment, we observed a significant increase in Golgi dispersion in PD1^+^TIM3^+^ T cells compared to PD1^−^TIM3^−^ (Fig. 5L). This correlated with our observation that PD1^+^TIM3^+^ terminally exhausted T cells had significantly lower expression of GM130 compared to PD1^−^TIM3^−^ T cells (Fig. 5M). Given our observation that H_2_S treatment protects antitumor T cells from oxidative stress, we next sought to determine whether oxidative stress plays a role in mediating Golgi stress. When Pmel T cells were treated with monensin to induce Golgi stress, a significant increase in ROS (2',7'-dichlorofluorescein diacetate, DCFDA) was observed, which was mitigated with H_2_S treatment (Fig. 5N). The changes in Golgi stress (quantified by GM130 levels) mediated by oxidative stress (using H_2_O_2_) were mitigated by H_2_S (Fig. 5O). Further, human T cells were also evaluated for Golgi dispersion by confocal microscopy, along with mitochondria staining to establish whether there is any spatial relationship between the Golgi and mitochondria. Compared to vehicle control, H_2_S-treated T cells had more compact Golgi organization. We also observed a close spatial relationship between the Golgi and mitochondria with areas of overlap between the two organelles (fig. S5E).

### Prdx4 regulates H_2_S-mediated inhibition of Golgi stress

**Fig. 6.**
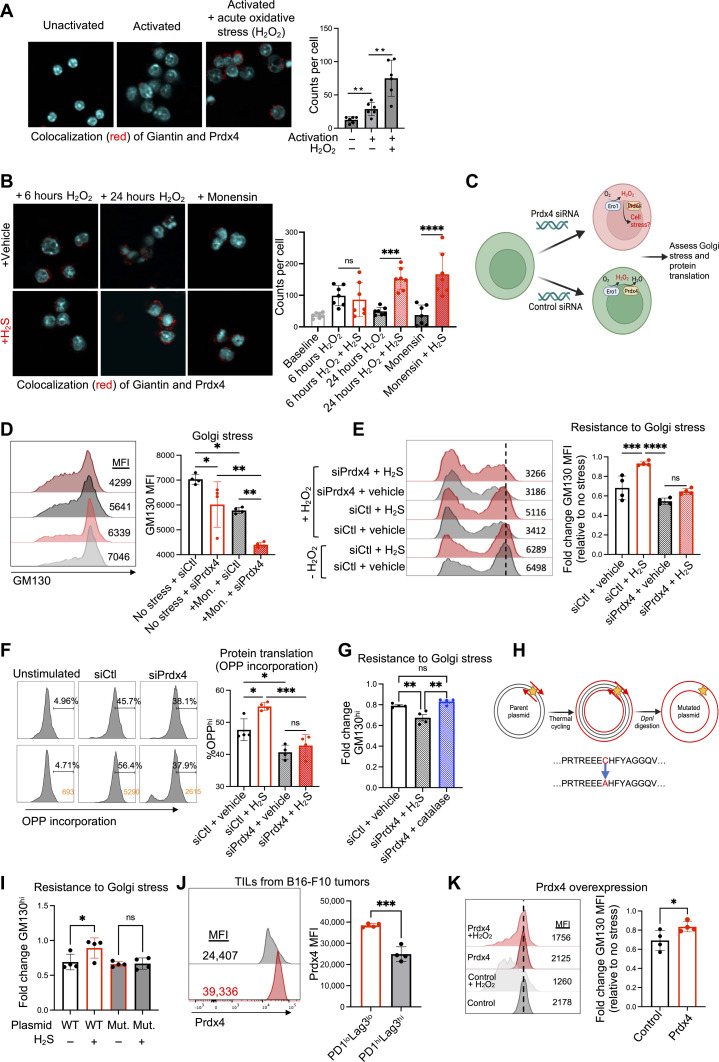
Prdx4 regulates H_2_S-mediated inhibition of Golgi stress. (**A**) Pmel T cells were activated for 3 days before treatment with vehicle or 50 mM H_2_O_2_ for 6 hours. Prdx4-Golgi colocalization was assessed by PLA using mouse anti-Giantin and rabbit anti-Prdx4 antibodies. (**B**) Activated Pmel T cells treated with vehicle or 50 mM H_2_O_2_ for 6 or 24 hours with monensin (1 mM). PLA was performed using anti-Giantin and anti-Prdx4 antibodies. (**C**) Experimental design using Prdx4-targeted siRNA to silence expression of Prdx4 in Pmel T cells. (**D**) GM130 expression assessed under control versus monensin (1 μM) conditions in T cells treated with control-siRNA or Prdx4-siRNA. (**E**) GM130 expression assessed under control versus 50 mM H_2_O_2_ conditions in T cells treated with control-siRNA or Prdx4-siRNA with or without 0.5 mM GYY4137. (**F**) Protein translation assessed in Pmel T cells activated with or without GYY4137 and treated with control-siRNA or Prdx4-siRNA. (**G**) GM130 expression in Pmel T cells treated with vehicle, GYY4137, or catalase (10 μg/ml). (**H** and **I**) Site-directed mutagenesis performed to mutate cysteine-54 of the Prdx4 gene. Prdx4-targeted shRNA was used to knock down expression of Prdx4 in Jurkat T cells, followed by transfection with plasmid containing wild-type or mutated Prdx4. (I) GM130 expression assessed after treatment with 50 mM H_2_O_2_ with or without GYY4137. (**J**) B16-F10 tumors were injected subcutaneously into flanks of Pmel mice and grown to ~100 mm^2^. TILs and T cells from tumor-draining lymph nodes were isolated via Ficoll spin followed by magnetic bead positive selection. FACS analysis of Prdx4 expression in TILs gated on PD1^lo^Lag3^lo^ versus PD1^hi^Lag3^hi^. (**K**) Lentiviral supernatant was generated using GFP-tagged control plasmid or GFP-tagged Prdx4-overexpression plasmid. Pmel T cells were transduced with control or Prdx4 lentivirus. Pmel T cells overexpressing Prdx4 and control T cells were treated with H_2_O_2_ (50 mM) followed by staining with anti-GM130. Data shown represent mean ± SEM and analyzed by two-sided Student’s *t* test or one-way ANOVA. ns, *P* > 0.05; *P ≤ 0.05; **P ≤ 0.01; ***P ≤ 0.001; ****P ≤ 0.0001.

Our proteomics screen (Fig. 5D) suggested that an increase in free thiols at cysteine 54 in Prdx4 is promoted by H_2_S treatment. Prdx4 is a key peroxiredoxin enzyme that is involved in regulating redox balance and oxidative stress within the ER. Given the delicate redox balance necessary for the native disulfide bonds, we postulated that a localized mechanism for the detection and elimination of ROS produced during the oxidative folding process may require Prdx4. Thus, we hypothesized that ER and Golgi network stress mitigated by H_2_S is mediated through the Prdx4 localization and activity in these organelles. First, we sought to establish whether Prdx4 also localizes within the Golgi apparatus in T cells using a proximity ligation assay (PLA), a tool that allows in situ detection of endogenous proteins with high specificity and sensitivity, with antibodies directed against Giantin (a conserved Golgi membrane protein) and Prdx4. We observed that Prdx4 localizes within the Golgi apparatus upon T cell activation (Fig. 6A). This localization was further increased upon induction of acute oxidative stress, suggesting a previously unidentified role of Prdx4 in responding to oxidative stress within the Golgi (Fig. 6A). Prolonged oxidative stress ultimately resulted in loss of Prdx4 localization within the Golgi, resembling a colocalization profile similar to cells treated with monensin to induce Golgi stress (Fig. 6B). Notably, this loss of colocalization was mitigated with H_2_S treatment (Fig. 6B).

Next, using siRNA targeting *Prdx4,* we knocked down expression of *Prdx4* in CD8^+^ Pmel T cells (Fig. 6C and fig. S6, A and B). We then subjected the cells to conditions of oxidative and Golgi stress in the presence or absence of H_2_S treatment. Of note, knockdown of *Prdx4* resulted in a significant decrease in GM130 expression (Fig. 6D). This decrease was further exacerbated with monensin treatment, demonstrating an increase in susceptibility to Golgi stress in the absence of Prdx4 (Fig. 6D). Then, to determine whether the protective effect of H_2_S on Golgi stress is dependent on the presence of Prdx4, we induced Golgi stress in the presence or absence of H_2_S with control (*siCtl*) and *Prdx4* knockdown (*siPrdx4*) T cells. Of note, while H_2_S prevented the decrease in GM130 expression in control T cells, this protective effect was absent in *Prdx4* knockdown T cells (Fig. 6E). A similar dependence on the presence of Prdx4 was observed for the effect of H_2_S on increasing protein translation, which was not observed upon H_2_S treatment in the *Prdx4* knockdown T cells (Fig. 6F). In accordance, the *Prdx4*-silenced T cells showed reduced production of effector cytokines (IFN-γ and TNFα) and cytolytic molecules (granzyme B and perforin) (fig. S6C).

Given the key role of Prdx4 in scavenging superoxide species and our previous findings indicating a role for oxidative stress (specifically H_2_O_2_) in disrupting the Golgi, we hypothesized that it is an organelle-specific H_2_O_2_ scavenging function of Prdx4 that is critical for its ability to protect against Golgi stress. To test this, we induced Golgi stress in control or Prdx4 knockdown T cells in the presence of H_2_S or the direct H_2_O_2_ scavenger catalase. As observed previously, the protective effect of H_2_S in mitigating Golgi stress was absent when Prdx4 was knocked down, but protection against Golgi stress could be rescued with the addition of catalase (Fig. 6G). Given the increase in free thiol at cysteine 54 in Prdx4 with H_2_S, we transfected Prdx4 knockout Jurkat cells with plasmids containing either wild-type Prdx4 or a version of Prdx4 mutated at the homologous human residue, C51A (Fig. 6H). The protective effect of H_2_S on reducing H_2_O_2_-induced Golgi stress was only observed in Jurkat cells expressing wild-type Prdx4 and not in the cells expressing the cysteine-mutated form of the protein (Fig. 6I). A similar Prdx4-dependent effect of H_2_S on reducing ER stress was observed Jurkat cells expressing either the normal or cysteine-mutated form of Prdx4 (fig. S6D).

To further establish the physiological relevance of T cell Prdx4 expression in the TME in vivo, we isolated TILs from B16-F10 tumors and assessed levels of Pdrx4 in PD1^hi^Lag3^hi^ terminally exhausted TILs versus PD1^lo^Lag3^lo^ TILs. Notably, we observed a significant decrease in Prdx4 expression in PD1^hi^Lag3^hi^ terminally exhausted TILs (Fig. 6J). In addition, we observed a significant decrease in Prdx4 expression in antigen-experienced TILs compared to antigen-experienced T cells in the tumor draining LNs, suggesting a suppressive effect of the TME on sustained Prdx4 expression (fig. S6E). To further verify the role of Prdx4 in sustaining T cell function and mitigating Golgi stress, lentivirus supernatant was generated to overexpress Prdx4 in activated Pmel T cells (fig. S6F). Pmel T cells overexpressing Prdx4 were more resistant to loss of GM130 compared to control Pmel T cells (Fig. 6K). Upon restimulation, Pmel T cells overexpressing Prdx4 produced more cytolytic cytokines compared to control Pmel T cells (fig. S6G). Collectively, these findings demonstrate a protective effect of H_2_S in reducing Golgi stress that is at least partially dependent on the H_2_O_2_-scavaging capacity of Prdx4.

### Golgi^hi^ cells exhibit enhanced functionality and stem-like features

**Fig. 7.**
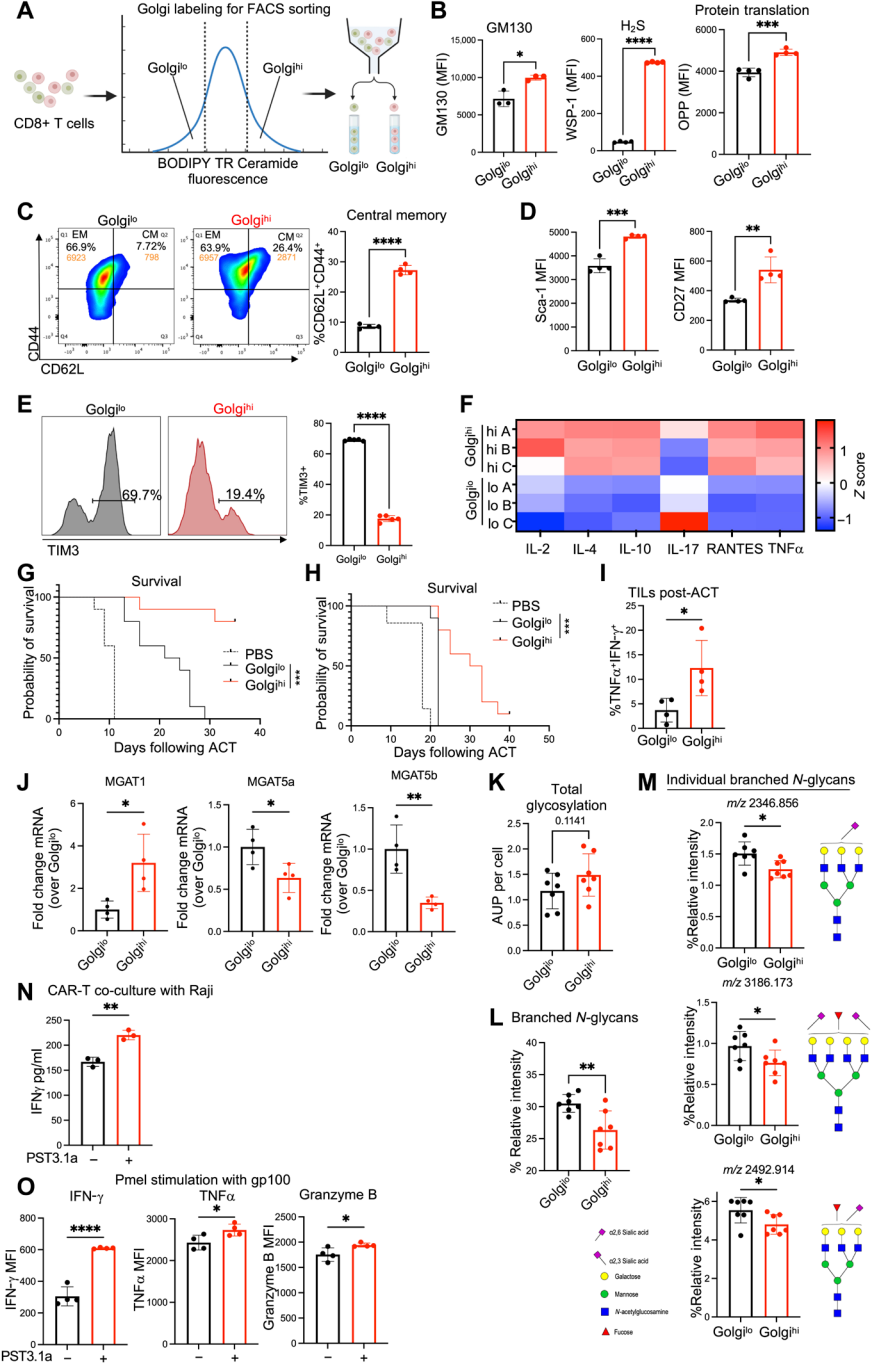
Golgi^hi^ cells have enhanced functionality and stem-like features. (**A** to **K**) Pmel T cells were activated for 3 days with or without GYY4137, then stained with BODIPY-TR Ceramide dye to label Golgi, followed by FACS-sorting into Golgi^hi^ (upper 30%) and Golgi^lo^ (lower 30%) based on BODIPY-TR Ceramide dye fluorescence. (B) FACS quantification of GM130 and H_2_S levels in Golgi^hi^ versus Golgi^lo^ cells. Sorted cells were restimulated (gp100 1 μg/ml) and analyzed for protein translation using the Click-iT Plus Protein Synthesis Kit (*n* = 4). (C) FACS quantification of CD62L^+^CD44^+^ populations in sorted cells (*n* = 4). (D) FACS quantification of Sca-1 and CD27 in sorted cells (*n* = 4). (E) FACS analysis of TIM3 expression in sorted cells after in vitro exhaustion assay (*n* = 5). (F) Sorted cells were restimulated (gp100 1 μg/ml) and the supernatant was collected for multiplex cytokine ELISA. Heatmap illustrating log10(fluorescence intensity) of cytokines for Golgi^lo^ versus Golgi^hi^ groups (*n* = 3). [(G) and (H)] Overall survival for (G) B16-F10 tumor-bearing C57BL/6 mice treated with FACS-sorted Pmel T cells (*n* = 10) and (H) Raji tumor-bearing NSG mice treated with FACS-sorted CD19-CAR-T cells (*n* = 10). (I) Sorted Pmel T cells were injected into B16F10-bearing mice. TILs isolated by Ficoll separation and CD8^+^ selection stimulated with gp100 followed by FACS analysis of TNFα and IFN-γ (*n* = 4). (J) qRT-PCR analysis of mRNA levels for glycosylation enzymes in Golgi^hi^ *versus* Golgi^lo^ human CD8^+^ T cells. (K) Golgi^hi^ versus Golgi^lo^ human CD8^+^ T cells analyzed using antibody-based platform with MALDI-IMS to assess N-linked glycosylation. (**L** and **M**) Levels of branched *N*-glycans with individual branched *N*-glycans that were significantly different between groups. (**N**) ELISA quantification of IFN-γ production after CD19-CAR-T cells were treated with vehicle or Phostine-PST3.1a (10 μM) and cocultured with Raji cells (*n* = 3). (**O**) Pmel T cells treated with vehicle or Phostine-PST3.1a before restimulation for FACS cytokine analysis (*n* = 4). Data shown represent mean ± SEM, analyzed by two-sided Student’s *t* test or one-way ANOVA. ns, *P* > 0.05; *P ≤ 0.05; **P ≤ 0.01; ***P ≤ 0.001; ****P ≤ 0.0001.

On the basis of our findings that T cells exposed to the TME exhibit increased Golgi stress, which correlated with a decrease in GM130 levels and a decrease in antitumor function, we sought to determine Golgi content itself would correlate with T cell antitumor function. Thus, activated CD8^+^ T cells were stained using a fluorescent dye to label the Golgi and were subsequently sorted into cells possessing high Golgi content (Golgi^hi^) and low Golgi content (Golgi^lo^) (Fig. 7A). We observed significantly increased levels of GM130, intracellular H_2_S, and protein translation in the Golgi^hi^ T cells upon restimulation with gp100 tumor antigen compared to the Golgi^lo^ T cells (Fig. 7B). The Golgi^hi^ subset also exhibited an increase in the Tcm phenotype, with Golgi^hi^ cells having a higher proportion of CD62L^+^CD44^+^ cells (Fig. 7C) and higher expression of Sca-1 and CD27 (Fig. 7D).

In the TME exhaustion assay, Golgi^lo^ cells had a significantly higher expression of TIM3 as compared to Golgi^hi^ cells, indicating a resistance to T cell exhaustion in the Golgi^hi^ subset (Fig. 7E). Functionally, Golgi^hi^ cells secreted more pro-inflammatory cytokines upon restimulation with cognate antigen, including IL-2, IL-4, RANTES, and TNFα (Fig. 7F). We observed that treating Golgi^lo^ T cells with H_2_S resulted in partial restoration of their effector functions (fig. S7A). Golgi^hi^ cells were also characterized by a significant increase in mitochondrial mass compared to Golgi^lo^ (fig. S7B). Similar to H_2_S-treated T cells, Golgi^hi^ T cells also displayed a significant increase in SRC, demonstrating a correlation between Golgi^hi^ cells and enhanced mitochondrial fitness (fig. S7C). Transmission electron microscopy analysis of Golgi^hi^ versus Golgi^lo^ cells revealed large, healthy-appearing mitochondria in Golgi^hi^ cells compared to small, dense mitochondria in the Golgi^lo^ cells (fig. S7D). Metabolomics analysis revealed significant differences in the metabolite profiles of Golgi^hi^ versus Golgi^lo^ cells (fig. S7E). Of note, several key metabolic pathways were found to be up-regulated in the Golgi^hi^ cells compared to Golgi^lo^ cells, including GSH metabolism, nicotinate/nicotinamide metabolism, and mitochondrial electron transport chain (fig. S7, F and G). Similar to H_2_S-treated T cells, Golgi^hi^ cells had a significantly higher NAD^+^/NADH ratio (fig. S7H).

We next sought to determine the antitumor capacity of Golgi^hi^ versus Golgi^lo^ cells, hypothesizing that Golgi^hi^ cells would exert superior tumor control when adoptively transferred into tumor-bearing hosts. We found that Golgi^hi^ Pmel T cells displayed significantly better tumor control (fig. S7I) and improved survival (Fig. 7G) in B16-F10–bearing C57BL/6 mice. In addition, when human CD19 CAR-T cells were sorted into Golgi^hi^ and Golgi^lo^ populations and adoptively transferred to the NSG mice engrafted with Raji tumor cells, the Golgi^hi^ CAR-T cells displayed superior control of lymphoma (fig. S7J) and prolonged host survival (Fig. 7H). Adoptively transferred Golgi^hi^ cells were detected at higher circulating frequencies after transfer (fig. S7K), exhibited reduced exhaustion and preserved Tcm phenotype (LAG3^lo^CD62^hi^CD27^hi^) (fig. S7L), and maintained superior effector function when isolated from the TME (Fig. 7I). When cocultured with Raji cells, Golgi^hi^ CAR-T cells also induced significantly more tumor cell death compared to the Golgi^lo^ CAR-T cells (fig. S7M) and produced more perforin and granzyme B (fig. S7N). These data confirm that sorting T cells based on the status of the Golgi apparatus identifies T cells with superior ability to control tumor cells.

To further identify the pathways that define the Golgi^hi^ versus Golgi^lo^ subsets, RNA-seq analysis was performed on human CD19 CAR-T cells, which revealed a district transcriptomic profile between the two subsets (fig. S7O). Fascinatingly, the top two up-regulated pathways in the Golgi^hi^ subset involved microtubule and tubulin binding (fig. S7P). Of note, previous studies have identified a critical role for Golgi proteins in coordinating with the microtubule organizing center to facilitate transportation of key signaling molecules to the immunological synapse (*46*). In addition, RNA-seq analysis revealed significant differences in levels of key enzymes involved in regulating *N*-glycan branching in the Golgi, including an increase in *MGAT1* (β1,6 *N*-acetylglucosaminyltransferase I, a negative regulator of *N*-glycan branching) and a decrease in *MGAT5A/B* (β1,6 *N*-acetylglucosaminyltransferase Va/Vb, the rate-limiting enzyme in *N*-glycan branching), which were validated by reverse transcription polymerase chain reaction (RT-PCR) (Fig. 7J). Given the critical role of the Golgi in posttranslationally modifying proteins via glycosylation, we profiled the N-linked glycosylation on Golgi^hi^ versus Golgi^lo^ T cells using a method published by Dressman *et al.* (*47*). While an overall increase in total glycosylation in Golgi^hi^ cells was observed, it was not statistically significant (Fig. 7K). However, a notable decrease in overall branched *N*-glycans was observed (Fig. 7, L and M), which correlated with the reduced expression of *Mgat5a/b*. Levels of branched *N*-glycans have been shown to be directly correlated with the T cell activation threshold in an Mgat5-dependent manner, while Mgat1 has been shown to paradoxically inhibit the activity of Mgat5, leading to decreased *N*-glycan branching (*48*–*50*). To further validate the importance of decreased Mgat5 activity and thus low *N*-glycan branching in the Golgi^hi^ subset, we treated activated CD8^+^ T cells with the Mgat5 inhibitor Phostine PST3.1a, a selective inhibitor of Mgat5 enzymatic activity, which has been shown to have antitumor activity in in vivo models of glioblastoma (*51*). As predicted, inhibition of Mgat5 activity in both human and murine T cells resulted in a more potent effector response upon encountering tumor antigen, as evidenced by an increase in IFN-γ production by CD19 CAR-T cells when cocultured with Raji tumor cells (Fig. 7N) and an increase in cytolytic cytokine production by Pmel T cells upon restimulation with gp100 peptide (Fig. 7O). Thus, a role for the Golgi in modulating protein glycosylation and rendering a robust antitumor phenotype to T cells could be a key factor in determining immunotherapeutic outcomes.

## DISCUSSION

The cumulative role of cellular organelles in shaping the life and function of a cell has been long acknowledged (*52*). While each organelle plays a specific role in the growth and development of T cells, numerous studies have thus far focused on targeting mitochondria-, ER-, or lysosome-related pathways to improve the antitumor T cell immune response. Strategies mitigating stress in these organelles have shown to improve T cell fitness and enhance tumor control. Increasing evidence suggests that the Golgi apparatus also plays a crucial function in sensing and integrating external and internal cues to promote cellular homeostasis. The Golgi apparatus is essential for maintaining normal cell physiology because it supports cell survival, promoting cell proliferation, and facilitating cell-cell communication and migration. These roles are partly influenced by established Golgi functions, such as posttranslational modifications, lipid production, intracellular trafficking, and protein secretion (*19*). Because intracellular organelles are tightly regulated under various stress conditions, we hypothesized that Golgi apparatus disruption under oxidative stress could alter lipid and protein modification, packaging, and transport, resulting in suboptimal antitumor T cell function.

Disruption of Golgi architecture and functions, termed Golgi stress, has been previously shown to alter redox balance and affect cell survival (*43*). Golgi stress inducers, including monensin and brefeldin A, have been widely shown to impair Golgi structure and function. These Golgi stressors have been shown to up-regulate CSE and endogenous H_2_S generation, whereas inhibition of the CSE/H_2_S system results in increased susceptibility to Golgi stress (*17*). Thus, we hypothesized that treating T cells with exogenous H_2_S would overcome Golgi stress and restore Golgi apparatus function to enhance antitumor T cell response. The role of H_2_S in biological processes has increasingly become the focus of research in recent years. A particular focus has been on the cytoprotective and antioxidant properties that H_2_S appears to have in cells that are exposed to high levels of oxidative stress (*53*–*62*). We found that with traditional T cell activation methods, T cells markedly up-regulate H_2_S production; however, as T cells continue to proliferate, this initial increase in H_2_S returns to baseline levels. These results initially suggested to us that H_2_S signaling may play an important role in supporting T cell activation and function and that sustained H_2_S signaling could produce robust antitumor T cells. Antitumor T cells expanded with exogenous H_2_S or overexpressing CBS to increase endogenous H_2_S production in T cells leads to high levels of cytolytic cytokines and sustained levels of protein translation upon TCR stimulation.

Our data demonstrate that H_2_S is an important immunomodulatory signaling molecule that can be used to alter multiple factors in T cells to enhance their antitumor capacity and that this approach can be used to program TILs and genetically modify T cells with potent antitumor phenotype. Treating T cells with H_2_S donors or increasing endogenous production of H_2_S supports previously established signatures of robust antitumor T cells, such as enhanced stemness, increased mitochondrial function, and reduced susceptibility to oxidative stress and ER stress upon chronic antigen stimulation. Our data also highlight that the increased Tcm phenotype observed in H_2_S-treated T cells can be attributed to enhanced NAD^+^ levels, through the NAD^+^-Sirt1-Foxo1 axis (*9*, *63*). Foxo1 has also been recently shown to be important for determining CAR-T cell memory phenotype and function (*36*, *37*). Thus, it is likely that H_2_S acts at multiple levels to render a stress-free Tcm phenotype that results in improved persistence in vivo upon adoptive transfer to bring improved tumor control.

In other cell types, H_2_S has been shown to reduce ER stress, particularly in the context of oxidative stress (*64*, *65*). We observed a similar effect of H_2_S in T cells, both in reducing overall oxidative stress and in preventing ER stress upon chronic antigen stimulation. Our data show that dysfunction within the Golgi apparatus is another critical factor that needs to be considered when generating tumor-reactive T cells for adoptive therapies and that H_2_S treatment during the expansion process can be used for effective programming. However, it must be noted that given the interdependence of organelle function in shaping the cellular response, a limitation of this study remains in determining the sole role of Golgi stress in altering the immune response when mitochondria or ER stress is also affected. Nonetheless, these findings offer insight into the role of H_2_S signaling in regulating both ER and Golgi network stress in T cells and offer previously unidentified therapeutic strategies for improving antitumor T cell response.

While the ER and its associated ribosomes are responsible for synthesizing and folding proteins, the Golgi apparatus is a closely associated organelle responsible for further modification and sorting of synthesized proteins received from the ER. We were thus particularly interested in the status of the Golgi apparatus in T cells, given the importance of Golgi processing of the secreted factors that are required for T cell effector function. Similar to ER stress, multiple studies have shown that cells can also experience Golgi stress, characterized as a fragmentation of the Golgi apparatus and an inability to process proteins properly (*66*–*68*). Studies have recently shown that H_2_S is an important protective regulator of Golgi stress (*17*). We found that Golgi stress was a characteristic of exhausted T cells and that treatment with H_2_S could reduce Golgi stress in antitumor T cells. Our data also demonstrate an important role for the thiol-specific peroxidase Prdx4 in regulating ER and Golgi stress in antitumor T cells. Prdx4 reactive cysteines are particularly susceptible to oxidation, rendering Prdx4 inactive when H_2_O_2_ levels are high (*69*). Strategies to target and selectively reduce the functional cysteine residues of Prdx4 and other molecules that regulate Golgi function will have the potential for high translational value to optimize immunotherapy. These findings identify Golgi stress as a previously unidentified therapeutic target in cancer immunotherapy and identify H_2_S-based therapy as a potential strategy for mitigating Golgi stress to enhance antitumor immunity.

Further, it is of substantial interest to the scientific and medical communities to identify simple phenotypic attributes of potent antitumor T cells. For example, Sukumar *et al.* (*8*) earlier demonstrated that T cells with low mitochondrial membrane potential as measured with TMRM dye had robust antitumor capacity and that T cells with low mitochondrial membrane potential could be sorted and used for ACT to produce durable tumor control. Similarly, we show that T cells with high and low Golgi mass have distinct functionality profiles and that sorting on Golgi^hi^ cells produces a subset of T cells with superior ability to control tumors. Whether it is increased expression of key cell signaling molecules in Golgi^hi^ fraction resulting from asymmetric cell division and distribution that contributes to long-term maintenance of T cell function and control (*70*) or whether it is reduced activity of Mgat5-dependent *N*-glycan branching that lowers T cell threshold of activation and leads to increased functionality (*49*) will need to be dissected in the future. Regardless, this approach to cell selection will likely be broadly applicable to multiple forms of ACT for treating cancer, including TIL and CAR-T therapy.

Overall, it is of critical importance to continue to identify novel therapeutic strategies for enhancing the ability of the immune system to control and eliminate tumors. An improved understanding of regulation of the T cell immune response at the organelle level can help devise effective antitumor therapies focused on reducing organelle stress, limiting organelle damage, improving inter-organelle cross-talk, and restoring organelle homeostasis, and could be useful to improve immunotherapy options. Ultimately, we believe that H_2_S signaling plays a key role in immune regulation at multiple levels, including reduction of Golgi stress in antitumor T cells, which can be used to boost antitumor immunotherapeutic strategies.

## MATERIALS AND METHODS

### Materials availability

This study did not generate new or unique reagents.

### Data and code availability

Single-cell RNA-seq data have been deposited at Gene Expression Omnibus (GEO) and are publicly available as of the date of publication. The mass spectrometry proteomics data have been deposited to the ProteomeXchange Consortium via the Proteomics Identification Database (PRIDE) partner repository. Accession numbers are listed in the key resources table. This paper does not report original code.

### Mice

C57BL/6, B6-Rag^−/−^, Pmel, NSG, and Cbs^−/−^ mice were obtained from the Jackson Laboratory (Bar Harbor, ME). Animals were maintained in pathogen-free facilities, and experimental procedures were approved by the Institutional Animal Care and Use Committees of Medical University of South Carolina, Charleston (approval no. IACUC-2018-00628-1). For tumor experiments, an equal number of age- and gender-matched (both male and female) mice were randomly assigned for the experiments when they were between 8 and 10 weeks old. No influence of sex on the result of the studies was observed.

### Cell lines

B16-F10 and Jurkat cells were obtained from the American Type Culture Collection (ATCC), suggesting male origin. Raji cells (ATCC no. CCL-86) were a gift from M. Nishimura (Loyola University Chicago).

### Generation of Prdx4 knockout and Prdx4 mutant cells

Jurkat cells were transduced with human Prdx4 shRNA lentiviral particles expressing a puromycin resistance gene. Puromycin was added to the culture media to selectively expand the transduced Jurkat cells. Prdx4 knockdown was confirmed by RT-PCR and Western blot analysis. To generate the Prdx4 mutant plasmid, primers were designed based on the coding sequence of the canonical gene of interest (Prdx4). The coding sequence was converted into the amino acid codon sequence using Expasy to mutate the amino acid of interest. Roughly 15 to 20 amino acids upstream and downstream of the mutated codon were selected, and the New England Biolabs Tm Calculator was used to adjust the primer length, projected annealing temperature, and GC content. The Harvard Reverse Complement Tool was used to produce the reverse primer sequence. Primers were ordered from Integrated DNA Technologies, including 5′-phosphorylation for plasmid ligation. The template plasmid (containing the wild-type gene of interest for mutation) and the primers were then used with the QuikChange XL Site-Directed Mutagenesis kit (Agilent Technologies, no. 200516) per the manufacturer’s instructions. The successful mutation was confirmed via sequencing of the plasmid (Genewiz, Azenta Life Sciences).

### Overexpression of Prdx4

To generate the lentiviral particle containing prdx4 plasmid, 293T Lenti-X cells were seeded in complete Dulbecco’s Modified Eagle’s Medium (DMEM) overnight in a 10-cm tissue culture plate. The next day, 4 hours before transfection, the cells were treated with 25 μM chloroquine. Following chloroquine treatment, cells were transfected with 10 μg of either Prdx4 plasmid or mock plasmid and 7.5 μg of psPAX2 packaging plasmid and 2.5 μg of pMD2.G envelope plasmid through Lipofectamine 3000 according to the manufacturer’s protocol. The next day, the medium was replaced with fresh complete DMEM and allowed to grow for 24 hours. The next day, media containing virus particles were collected and filtered through a 0.45-μm syringe filter. Supernatants containing viral particles of either mock or Prdx4 insert were diluted at 1:1 ratio with fresh complete Iscove’s Modified Dulbecco’s Medium (IMDM) before mouse T cell transduction. T cells were collected from spleen of healthy Pmel mouse, and 1 × 10^6^ cells/ml were transduced with diluted viral supernatant by spinoculation method in the presence of protransdusin at 2000 rcf at 32°C for 2 hours. Twenty-four hours later, cells were collected, washed, and checked for green fluorescent protein (GFP) expression before use for further experimental analysis.

### T cell differentiation

Naïve total T cells were purified from the total splenocytes of 6- to 9-week-old C57BL/6 mice, first by incubating the cells with biotinylated anti-CD19, anti-Gr1, anti-mouse ter-119, anti-CD11b, anti-CD11c, anti-NK1.1, anti-CD25, and anti-CD105 (Cell Signaling Technology), followed by negative selection with streptavidin magnetic particles (BD Biosciences). Purified T cells were then activated with soluble anti-CD3 (1 μg/ml) and anti-CD28 (1 μg/ml) in the presence of IL-2 (100 IU/ml). Total splenocytes from 6- to 9-week-old Pmel transgenic mice (bearing class I restricted CD8^+^ T cells) were activated with gp100 melanoma antigen (1 μg/ml) in the presence of IL-2 (100 IU/ml). Within experiments, mice were age and sex matched. T cells were cultured in IMDM media supplemented with 10% fetal bovine serum (FBS), 4 mM l-glutamine, penicillin (100 U/ml), streptomycin (100 μg/ml), 55 μM β-mercaptoethanol under 7% CO_2_, and atmospheric oxygen at 37°C in a humidified incubator. T cells were restimulated to evaluate intracellular cytokines by flow cytometry either with phorbol myristate acetate (PMA)/ionomycin for 4 hours or soluble anti-CD3 (1 μg/ml) and anti-CD28 (1 μg/ml) or with gp100 melanoma antigen for 6 hours in the presence of Golgi inhibitors. In some experiments, in vitro differentiated T cells were treated with either the vehicle control or H_2_S donor GYY4137 (0.5 mM).

### Retroviral transduction

For CD19-CAR-T generation, human peripheral blood mononuclear cells (PBMCs) were obtained from healthy donors by Ficoll gradient spin and activated for 3 days with soluble anti-CD3 antibody (Okt-3, 1 μg/ml). For Cbs overexpression, freshly isolated Pmel T cells were activated with gp100 peptide (1 μg/ml) for 3 days. CD19-CAR-T and CBS-CD19-CAR-T viral supernatant was generously gifted by M. Nishimura (Loyola University Chicago). Cbs viral supernant for Pmel transduction was generated using Cbs human tagged ORF clone (Origene no. RC201755L4). After 3 days of activation, T cells were plated at a concentration of 2 × 10^6^ cells/ml in complete media onto non–tissue culture–treated 24-well plates (USA Scientific) coated with Retronectin. Viral supernatant (1 ml) was added on top of the T cells, and the plate was spun at 2000*g* for 2 hours and 32°C. After spinning, 1 ml of media was removed and replaced with fresh media containing IL-2 (200 IU/ml) before the cells were incubated overnight. The cells were collected, washed, and plated the following day for use in further experiments.

### Adoptive T cell protocol

B16-F10 (0.3 × 10^6^) melanoma tumor cells were injected subcutaneously into the left flank of 8- to 10-week-old C57BL/6 or Rag^−/−^ mice. After tumor establishment, recipient mice were injected intraperitoneally (i.p.) with cyclophosphamide (4 mg per mouse) before adoptively transferring intravenously either Pmel, Pmel-Cbs^td^, or TILs (1 × 10^6^). After adoptive T cell transfer, recipient mice were given IL-2 (50,000 U per mouse, i.p.) for three consecutive days. Raji cells (0.5 × 10^6^) were injected subcutaneously into the left flank of 8- to 10-week-old NSG mice. After tumor establishment, CD19-CAR-T cells (5 × 10^6^) were adoptively transferred intravenously. After adoptive T cell transfer, recipient mice were given IL-2 (50,000 U per mouse, i.p.) for three consecutive days. For all tumor control experiments, mice were randomly assigned to treatment groups and labeled using coded ear punch. Tumor measurements were then conducted in a blinded fashion until final analysis.

### In vitro TME exhaustion assay

B16-F10 (0.3 × 10^6^) melanoma tumor cells were injected subcutaneously into the left flank of 8- to 10-week-old C57BL/6 mice. Once the tumors reached a size of approximately 150 mm^2^_,_ the mice were euthanized and the tumors were removed. The tumors were then processed into single-cell suspension using a mouse tumor dissociation kit (Miltenyi Biotec, no. 130-096-730). The tumor cell suspension was then plated in six-well plates with IMDM media supplemented with 10% FBS, penicillin (100 U/ml), streptomycin (100 μg/ml), and 55 μM β-mercaptoethanol under 7% CO_2_, and atmospheric oxygen at 37°C in a humidified incubator. After 5 days of culture without changing the media, the supernatant was removed from the wells and spun down to remove any tumor cells. Three-day activated Pmel T cells were then resuspended in the tumor supernatant along with gp100 antigen (0.1 μg/ml). To promote chronic antigen stimulation, gp100 (0.1 μg/ml) was added every day for 4 days. After 4 days of culture with tumor supernatant and chronic stimulation, the T cells were removed and analyzed for expression of exhaustion markers and functional assays.

### Flow cytometry

Staining for cell surface markers was performed by incubating cells with the antibody at 1:200 dilutions in FACS buffer [0.1% BSA in phosphate-buffered saline (PBS)] for 30 min at 4°C. For intracellular cytoplasmic proteins, surface markers were stained before fixation/permeabilization (BD Cytofix/Cytoperm Kit, BD Biosciences, San Jose, CA). For staining of transcription factors, cells were stained with surface markers and fixed/permeabilized with a FoxP3 staining buffer set (eBioscience, San Diego, CA). For Cbs, pIRE1α, pPERK, and ATF4 intracellular staining, surface markers were stained before fixation/permeabilization, followed by primary unconjugated antibody staining and subsequent incubation with fluorochrome-conjugated secondary antibody (Jackson ImmunoResearch Laboratories, West Grove, PA). In addition, MitoTracker Red (Cell Signaling Technology no. 9082), LIVE/DEAD Fixable Yellow Dead Cell Stain Kit (Invitrogen no. L34959), DCFDA dye (Abcam no. ab113851), and WSP-1 dye (MCE no. HY-124409) were used to evaluate mitochondrial mass, cell viability, cellular ROS, and H_2_S production, respectively, following the manufacturer’s protocol. Samples were acquired on LSRFortessa and analyzed with FlowJo software (Tree Star, OR).

### Immunoblotting

For evaluation of the protein level, cell pellets were washed in PBS and lysed in radioimmunoprecipitation assay buffer (Thermo Fisher Scientific, Waltham, MA), including protease/phosphatase inhibitors, vortexed, and incubated for 20 min on ice. Cell lysates were then centrifuged at 12,000 rpm for 15 min at 4°C. The supernatants were collected, and proteins were quantified with a BCA protein assay kit (Thermo Fisher Scientific, Waltham, MA). For immunoblot analyses, 20 μg of protein lysates per sample was denatured in 4× Loading dye and boiled using a heating block at 95°C for 10 min before loading to SDS gradient gels 4 to 20% (Bio-Rad Criterion, 1-hour runs). Gels were semidry transferred onto polyvinylidene difluoride, and the membranes were blocked with 3% milk in 0.1% tris-buffered saline with tween 20 (TBST). Next, the membrane was probed with the following primary antibodies: anti-Prdx4 (Proteintech, 10703-1-AP), anti-eIF2α (Cell Signaling Technology, no. 9722), or anti–β-actin (Signaling Technology, no. 4967L) overnight at 4°C followed by 1-hour incubation with horseradish peroxidase–conjugated secondary antibody (Cell Signaling Technology, Danvers, MA) and using a Clarity Western ECL Substrate (Bio-Rad, Hercules, CA).

### Real-time quantitative PCR

Total RNA was extracted from pellets of the indicated T cell subsets (2 × 10^6^ cells) using TRIzol reagent (Life Technologies, Grand Island, NY). cDNA was generated from 1 μg of total RNA using the iScript cDNA Synthesis Kit (Bio-Rad, Hercules, CA). SYBR Green incorporation quantitative real-time PCR was performed using an SYBR Green mix (Bio-Rad, Hercules, CA) in the CFX96 Detection System (Bio-Rad, Hercules, CA). The expression of different genes was quantified relative to Actb. For RT-PCR arrays, RT^2^ Profiler PCR Arrays (Qiagen) were used according to the manufacturer’s instructions.

### RNA-seq analysis

Cells were immediately pelleted by centrifugation at 4°C and resuspended in 1 ml of TRIzol. RNA concentration was measured using a NanoDrop 8000. RNA quality was assessed using an Agilent 4200 TapeStation and RINe values ranged from 9.7 to 10. Total RNA (250 ng) was used in the construction of libraries with the New England Biolabs NEBNext Poly(A) mRNA Magnetic Isolation Module (catalog no. 7490L) and Ultra II Directional RNA Library Prep Kit for Illumina (catalog no. 7760L) according to the manufacturer’s instructions. Dual-indexed libraries were pooled to and sequenced at VANTAGE (Vanderbilt University Medical Center) on an Illumina NovaSeq 6000 (S4 flow cell) to a depth of approximately 25 million paired-end 150-bp reads per library. Reads were aligned to the mouse mm10 reference genome using STAR (v2.7.1a). Only uniquely mapped reads were retained for further analyses. Quality control metrics were assessed by the Picard tool (http://broadinstitute.github.io/picard/). Gencode annotation for mm10 (version M25) was used as reference alignment annotation and downstream quantification. Gene level expression was calculated using featureCounts (v2.0.1). Counts were calculated based on protein-coding genes from the annotation file. Counts were normalized using counts per million reads (CPM). Genes with no reads in either Control or Treated samples were removed. To infer potential experimental confounders, we calculated surrogate variables using the sva package in R. Differential expression analysis was performed in R using DESeq2 (v1.34) with the following model: gene expression ~ Treatment + nSVs. We estimated log_2_ fold changes and *P* values. *P* values were adjusted for multiple comparisons using a Benjamini-Hochberg correction [false discovery rate (FDR)]. Differentially expressed genes were considered for FDR < 0.05. Mouse Gene ID was translated into Human Gene ID using the biomaRt package in R. The functional annotation of differentially expressed genes was performed using clusterProfiler (v4.2). A Benjamini-Hochberg FDR (FDR < 0.05) was applied as a multiple comparison adjustment.

### Transmission electron microscopy

The cells were pelletized and fixed in 2% phosphate-buffered glutaraldehyde for 1 hour. Next, the pellets were rinsed in 0.1 M phosphate-buffered rinse and then postfixed in 2% aqueous osmium tetroxide for 1 hour. After rinsing in distilled water, the pellets were dehydrated through a series of graded ethyl alcohol; 50% ethanol (EtOH) for 15 min, 70% EtOH for 15 min, 95% EtOH for 15 min, and lastly twice with 100% EtOH for 15 min each. The dehydrant was removed using the intermediate fluid, propylene oxide, one change of 10 min each. Next, the pellets were infiltrated with a 1:1 solution of propylene oxide and Embed 812 (Electron Microscopy Sciences, Ft. Washington, PA) for 1 hour. The infiltration was continued using a 1:2 solution of propylene oxide and Embed 812 overnight. The pellets were embedded in Embed812 the following day and polymerized in a 60°C oven for 48 hours. Preliminary 1/2-μm sections were cut and stained with Toluidine Blue and examined using a light microscope. Then, with the cell types identified, the 70-nm thin sections were cut and stained with uranyl acetate and lead citrate and allowed to dry. The sections were viewed on the JEOL 1010, and images were taken with a Hamamatsu electron microscope camera.

### Confocal microscopy

Alexa Fluor 488 (anti-Giantin) and Alexa Fluor 647 (anti-TOMM20) fluorescence were imaged in a Zeiss LSM 880 NLO inverted laser scanning confocal microscope (Thornwood, NY) using a 63× 1.4 numerical aperture plan-apochromat oil immersion lens. Alexa Fluor 488 and Alexa Fluor 647 were excited at 488 and 633 nm, respectively. Emitted light was detected with an Airyscan super-resolution detector at BP 495 to 550 nm for Giantin label and LP 654 nm for TOMM20. Z-stack Airyscan images were processed using the Huygens Professional deconvolution and image analysis software (Scientific Volume Imaging, The Netherlands). After images were deconvolved using Huygens’ Deconvolution Express (Standard Profile) that determines optimal parameters, we performed three-dimensional (3D) surface rendering of deconvolved images with watershed augmentation using Huygens’ Surface Renderer.

### Metabolomics

Different metabolites’ intracellular levels were determined by performing comprehensive hydrophilic metabolites analysis using an Liquid chromatography–mass spectrometry (LC–MS) platform (Metabolomics Core Facility, Northwestern University). Data were then analyzed using MetaboAnalyst software. Samples were loaded equivalently across the platform and normalized to Bradford values before statistical analysis.

### Extracellular flux assays

Oxygen consumption rate (OCR) and extracellular acidification rate (ECAR) were determined using the Seahorse Xfe96 analyzer (Agilent Technologies, Santa Clara, CA). Briefly, T cells (0.5 × 10^6^ per well) were plated on a Cell-Tak coated Seahorse culture plate for 30 min. OCR, a measure of OXPHOS, was analyzed under basal condition, or in response to 1.0 μM oligomycin, 1.0 μM fluoro-carbonyl cyanide phenylhydrazone (FCCP), and 2 μM rotenone, plus 100 nM antimycin A. ECAR, a measure of glycolysis, was measured under basal conditions and in response to glucose (5.5 mM), oligomycin (1.0 μM), and 2-deoxyglucose (100 mM). All reagents were purchased from Sigma-Aldrich (St. Louis, MO).

### Protein translation assay

Pmel T cells were restimulated with gp100 peptide (0.1 μg/ml), and the Click-iT Plus OPP Alexa Fluor 647 Protein Synthesis Assay Kit (Thermo Fisher Scientific no. C10458) was used to measure protein translation using flow cytometry following the manufacturer’s protocol.

### Multispectral imaging flow cytometry analysis

T cells were stained with conjugated antibodies for surface markers as described in the figure legends followed by fixation and permeabilization with BD Cytofix/Cytoperm Kit (BD Biosciences, 554722). The Golgi was labeled using anti-Giantin antibody (Abcam, ab80864) as the primary antibody followed by secondary antibody staining with anti-rabbit immunoglobulin G (IgG) conjugated to Alexa Fluor 488 (ab150077). The cells were then imaged using ImageStreamX mark II imaging flow cytometer and analyzed using IDEAS 6.2. Spectral overlap was compensated for using single-stain controls. Image analysis for Golgi area was performed using the protocol established by Eisenberg-Lerner *et al.* (*44*). First, cells were gated on single cells using the area and aspect ratio features and then gated on focused cells using the Gradient RMS feature. Golgi area was calculated using the anti-Giantin fluorescence signaling, using the Threshold_50 mask that includes the 50% highest-intensity pixels of the Golgi staining, with a mask defined as Area_Threshold_50 considered as the Golgi area to compare between the relevant conditions.

### Proximity ligation assay

PLAs were performed using NaveniFlex Cell MR RED (Cayman, no. 39505) according to the manufacturer’s instructions. Anti-Prdx4 (Proteintech, 10703-1-AP) and anti-Giantin (Abcam, ab37266) were used as primary antibodies. T cells were fixed with 4% PFA for 15 min then permeabilized with 0.1% Triton X-100 for another 15 min at room temperature. PLA blocking agent was then used to block nonspecific binding, and the cells were then incubated overnight in the presence of antibodies of interest. After washing, secondary PLA probes conjugated to oligonucleotides were added to the cells, then a ligase was added to the samples to ligate the oligonucleotides that are in close proximity. DNA rolling-circle amplification was then performed using the PLA polymerase in the presence of fluorescence-bound oligonucleotide probes, which then yielded a fluorescent signal only where the two targets of interest are interacting in close proximity. Cells were imaged using the Olympus FV10i laser scanning confocal microscope. Signal quantification was performed using the Duolink in Situ Image Tool software.

### Isolation of tumor-infiltrating T cells

To obtain tumor-infiltrating T cells from subcutaneously established solid B16-F10 melanoma-bearing mice, tumors were excised, chopped finely using tweezers and scissors, and then digested with collagenase type IV (2 mg/ml; Stemcell Technologies, Vancouver, BC) for 45 min. The tumors were filtered through 70 cell strainers (BD Biosciences, San Jose, CA). The cell suspension was washed in culture medium twice by centrifugation at 1500 rpm for 10 min at 4°C. After the second wash, the cells were resuspended in 6 ml of PBS and layered carefully over 3 ml of Ficoll-Paque (GE Healthcare) followed by centrifugation at 1500 rpm for 30 min at room temperature. The enriched TILs obtained at the interface as a thin buffy layer were washed with PBS twice and lastly resuspended in FACS staining buffer for further staining procedures.

### Generation of bone marrow–derived dendritic cells

To obtain bone marrow, mice were euthanized by CO_2_ inhalation. Femoral bones were removed, and all remaining tissue was dissected off the bone. The ends of each bone were cut off and the bone marrow was flushed from the center of the bone. Bone marrow cells were then cultured on non–tissue culture–treated six-well plates in complete media for 7 days with granulocyte-macrophage colony-stimulating factor (GM-CSF; 10 ng/ml) and IL-4 (10 ng/ml) to generate dendritic cells. The medium was changed every 2 days. Floating cells were removed, and the loosely adherent cells were considered to be dendritic cells. FACS analysis was then performed to confirm the successful generation of dendritic cells.

### N-linked glycosylation profiling of T cells by mass spectrometry imaging

Antibody-based analysis of N-linked glycosylation was performed as fully described by Dressman *et al.* (*47*, *71*) In brief, amine-reactive slides were coated with antibodies at 200 ng per 1.5 μl spot and incubated at room temperature for 1 hour in a preheated humidity chamber. Bound antibodies were washed with 0.1% octyl-β-d-glucopyranoside in PBS (PBS-OGS) for 1 min, followed by blocking in 100 mM ammonium bicarbonate solution (pH 8) for 30 min. Antibodies were then deglycosylated by adding 100 μl of PNGase F PRIME (10 μg/ml) diluted in high-performance liquid chromatography (HPLC)-grade water into each well and placed back into the humidity chamber and incubated at 37°C for 2 hours. Following deglycosylation, antibody arrays were washed with PBS-OGS (3 min × 3) with gentle shaking followed by PBS washes (3×) and a water wash (1 min). T cells were washed (3×) in FACS buffer and resuspended in FACS buffer. Cell suspension (100 μl) was added to each well. Cell capture was performed at 4°C, shaking at 250 rpm for 1 hour. The 24-well module was then removed and the slide was placed in a slide mailer containing 10% neutral buffered formalin for 20 min. After 20 min, the slide was removed and placed in PBS at room temperature for up to 1 week before further processing. Sialic acid stabilization and derivatization were performed via a slide-based sequential amidation-amidation reaction with dimethylamine and propargylamine, termed AAXL (amidation-alkyne Xtra linker). To release *N*-glycans from captured cells, PNGase F Prime (0.1 μg/μl in HPLC water) was sprayed onto the slide using an M5 TM-Sprayer (HTX Technologies). Slides were incubated for 2 hours at 37°C in a preheated humidity chamber. Matrix-assisted laser desorption/ionization (MALDI) matrix α-cyano-4-hydroxycinamic acid (CHCA, 7 mg/ml in 50% acetonitrile/0.1% trifluoracetic acid) was sprayed using the same M5 TM-sprayer. Two passes of ammonium phosphate monobasic (5 mM) were sprayed across the slide to reduce matrix clustering and improve the signal. *N*-glycan imaging was conducted using a timsTOF-flex MALDI-Quadrupole Time-of-Flight (Q-TOF) mass spectrometer (Bruker) operated in positive ion mode at a mass/charge ratio (*m*/*z*) range of 700 to 4000. Images were collected using a SmartBeam 3D laser that operated at 10,000 Hz using the M5 small smart beam setting at a laser spot size of 100 μm run at a raster of 150 μm. Six hundred laser shots per pixel were collected with an ion transfer time of 120 μs, a prepulse storage of 25 μs, a collision radio frequency of 4000 Vpp, a multipole radio frequency of 500 Vpp, and a collision cell energy of 25 eV.

### Proteome-wide analysis of reactive cysteine thiols by LC-MS/MS–based proteomics

Cells were treated with or without H_2_S for 3 days of activation followed by 3 days of expansion. For analysis of differentially reactive cysteine residues, the sample preparation and analysis were as described by van der Reest *et al.* (*72*) with minor modifications. Cells were lysed in freshly made 9 M urea, 50 mM tris (pH 8) buffer with Universal Nuclease (100 U/ml; Thermo Fisher Scientific, Pierce catalog no. 88702). To label free thiols, lysis buffer was supplemented with either 55 mM of stable isotope-labeled light (I^12^C_2_ONH_4_) or heavy (I^13^CD_2_^13^CONH_2_) iodoacetamide (Sigma-Aldrich, catalog no. 721328) for control or H_2_S-treated cells, respectively. Equal amounts of heavy or light labeled proteins from three biological replicates were combined and reducible thiols were reduced with 70 mM dithiothreitol for 45 min at 25°C. After diluting with 50 mM ammonium bicarbonate, newly released thiols were alkylated with 80 mM *N*-ethylmaleimide (NEM) and incubated 2 hours at 25°C. Proteins were precipitated by adding 6 volumes of cold acetone, incubated overnight at −20°C, then centrifuged at 16,000*g* for 15 min at 4°C. The pellets were dissolved in an 8 M urea in 50 mM ammonium bicarbonate and the BCA protein assay was repeated. The concentration of urea was diluted to <2 M with ammonium bicarbonate and the proteins were digested with 1:33 (enzyme: protein) of Lys-C for 2 hours at 25°C and subsequently with 1:33 trypsin for 16 hours at 37°C while mixing at 300 rpm. The resulting peptides (100 μg) from three combined samples were each fractionated into six fractions using the high-pH RP spin columns according to the manufacturer’s protocol (Thermo Fisher Scientific, Pierce catalog no. 84868). Eluted peptides were dried by vacuum centrifugation. Two-microgram aliquots of peptides from each fraction, obtained using ZipTips with 0.6 μl of C18 resin (Millipore, Burlington, MA catalog no. ZTC18S096), were analyzed by LC-MS/MS on an Easy-nLC 1200 coupled to a Orbitrap Fusion Lumos MS (Thermo Fisher Scientific, Waltham, MA). Pressure-loaded peptides were chromatographically separated on a 75 μm × 50 cm column (Acclaim PepMap RSLC C18, 2 μm, 100 Å Thermo Fisher Scientific catalog no. 164540) thermostated at 45°C with a gradient of 5 to 35% solvent B in 180 min (solvent A: 5% acetonitrile, 0.2% formic acid and solvent B: 80% acetonitrile, 0.2% formic acid) at 300 nl/min. Mass spectra were acquired in data-dependent mode with a 3-s cycle between each MS1 acquisition. The FTMS survey MS scan mass range was *m*/*z* 375 to 1575. A quadrupole isolation window of 1.6 was used for precursor ion selection. Tandem mass spectra (MS/MS) were acquired following higher-energy collisional dissociation of peptide precursors with 35% collision energy. Ions were detected in the orbitrap. The automatic gain control (AGC) target value was 4 × 10^5^ for the survey MS scan at a resolution of 60,000 at *m*/*z* 400. The AGC target value for the MS/MS scan was 5 × 10^4^ with a maximum injection time of 22 ms. Precursors with charge states 2 to 7 were selected for fragmentation. Dynamic exclusion was enabled with a repeat count of 1, an exclusion duration of 25 s, and 10 parts per million mass tolerance.

To control for changes in protein expression, an aliquot of each uncombined, labeled protein sample was analyzed using a label-free proteomic approach (MaxQuant LFQ). Proteins were digested as above, and peptides were analyzed using a U3000 nano LC system coupled to an Orbitrap Elite MS (Thermo Fisher Scientific). Peptides were loaded onto a C18 PepMap 100 (300 μm × 5 mm) trap column for 10 min at 30 μl/min of solvent A and separated using a gradient of 5 to 40% solvent B in 180 min at 200 nl/min with a 75 μm × 35 cm fused-silica column [ReproSil-Pur 120 C18 AQ 1.9 μm at 50°C (ESI Source Solutions, Woburn, MA)] packed in-house. Mass spectra were acquired in data-dependent mode using a top 10 method. Each FTMS survey scan was acquired with a mass range of *m*/*z* 400 to 1500 in the Orbitrap followed by acquisition of the tandem mass spectra in the ion trap. A normalized collision energy of 35% was used with a 10-s activation time. The minimal signal for triggering acquisition of MS/MS was 500. Dynamic exclusion was enabled with a repeat count of 1, a repeat duration of 30 s, and an exclusion duration of 180 s.

The combined biological replicate samples were searched in MaxQuant v2.0.1.0 (Max Planck Institute) using a reviewed mouse database containing 17,090 protein sequences downloaded from UniProt on 30 November 2021. A strategy similar to that used for Stable Isotope Labeling by Amino Acids in Cell Culture (SILAC) experiments was used to set up the search. The labels introduced during sample lysis [either carbamidomethylation of Cys with light iodoacetamide (L-IAA) or heavy iodoacetamide (H-IAA)] were created in Andromeda within the MaxQuant platform to obtain ratios of heavy/light for the combined samples. The search was set up with a multiplicity of 2 (L-IAA and H-IAA). A database of contaminants was included in the search and a maximum of 2 trypsin missed cleavages were allowed. Methionine oxidation was used as a variable modification and no fixed modifications were defined. A decoy database strategy was used as a threshold for identifications with an FDR of 0.01 at the spectrum, peptide, and protein levels. The minimum peptide length was 7 and a minimum ratio count of 2 was required for quantitation. Match between runs was enabled. The entries from the “peptides” text file were processed in Perseus v1.6.15.0 (Max Planck Institute). The peptide list was filtered to eliminate common contaminants, entries from the reversed, decoy database, and peptides that did not contain cysteine. The H/L normalized ratios calculated by MaxQuant were log_2_ transformed and median normalized. Entries were filtered to retain peptides with ratios in each of the three biological replicate experiments. A *t* test was performed to test the null hypothesis by comparing the ratios to zero. The threshold for change in reactive thiol status was a Benjamini-Hochberg adjusted *P* < 0.05.

For the global proteomic analysis, each of the labeled, uncombined samples (*n* = 6) were analyzed in triplicate. The database search was performed as above with the exception of using a multiplicity of 1 and the label-free quantitation algorithm. Methionine oxidation and protein N-terminal acetylation were used as variable modifications; no fixed modifications were defined because the cysteines were modified with two distinct reagents (L-IAA and H-IAA) during cell lysis and NEM during sample preparation. The protein groups text file was processed in Perseus v.1.6.15.0. The list of proteins was filtered to eliminate common contaminants and entries from the reversed, decoy database. The protein intensities were log_2_ transformed and filtered to retain proteins quantified in three biological replicate samples of either the H_2_S-treated or control group. Missing values were imputed from a normal distribution with a width of 0.3 downshifted by 1.8. A *t* test was performed to compare the mean log_2_ intensities of proteins from H_2_S treated with control. To correct for multiple hypothesis testing, a permutation-based FDR of 0.01 was used as the threshold for change. Peptides and proteins were annotated with GO terms (downloaded from UniProt on 19 October 2021) and Reactome Pathway Names (downloaded on 2 October 2020) using Perseus. For visualization of the data volcano plots of the log_2_ fold change in peptide or protein abundance versus the −log_10_ *P* value were generated in Perseus.

### Statistical analysis

All data reported are the arithmetic mean from at least three independent experiments performed in triplicate ±SD unless stated otherwise. The unpaired Student’s *t* test was used to evaluate the significance of differences observed between groups, accepting *P* < 0.05 as a threshold of significance. Data analyses were performed using the Prism software (GraphPad, San Diego, CA), except for tumor control experiments where all calculations were performed in RStudio version 2023.06.1 using R-4.1.3. For all in vivo experiments with survival outcomes as the primary outcomes, it is expected based on preliminary data that 70% of the mice in the control group will be euthanized by 4 weeks. For a treatment to be successful, it would be expected that only 10% would be euthanized. A sample size of nine mice per group provides 80% power to detect this difference with a two-sided α of 0.05 using a log-rank test. For all survival outcomes, Kaplan-Meier curves were used to display the results. Median survival time and the corresponding 95% confidence interval were calculated for each experimental condition. A log-rank test was used to compare the outcomes across experimental conditions. For all continuous outcomes, graphical displays (e.g., bar charts) were used to demonstrate patterns of the outcomes within and across experimental conditions. Normality and variance homogeneity assumptions were assessed, and appropriate data transformations were used. All continuous outcomes were measured longitudinally from the same animal. These measures were modeled using linear mixed-effects regression, including fixed effects for experimental condition, time, and their two-way interaction; subject-specific random effects were incorporated to account for the correlation among measures obtained from the same subject over time. Linear contrasts were used to conduct group comparisons at each time point for which three or more mice were alive in each treatment group. For a given time point, *P* values were adjusted for multiple comparisons using Holm’s method of correction. Statistical analyses were performed in a blinded fashion with the statisticians only having access to coded group identifiers without knowledge of the corresponding treatment groups.

### Key resources

Reagent information is listed in Table 1.

**Table 1.** Reagent information.

Resource	Source	Identifier
Antibodies		
APC/Cyanine7 anti-mouse CD8a antibody	BioLegend	100714
PE anti-mouse CD8a antibody	BioLegend	100708
PE anti-human CD3 antibody	BioLegend	34406
PE anti-mouse CD11c antibody	BioLegend	117308
PE anti-mouse CD38 antibody	BioLegend	102708
PE anti-mouse CD62L antibody	BioLegend	104407
PE anti-mouse CD223 (LAG-3) antibody	BioLegend	125207
Pacific Blue anti-mouse CD11c antibody	BioLegend	117322
Brilliant Violet 711 anti-mouse CD279 (PD-1) antibody	BioLegend	135231
Brilliant Violet 650 anti-human CD34 antibody	BioLegend	561539
Alexa Fluor 700 anti-mouse Ly-6A/E (Sca-1) antibody	BioLegend	108142
Pacific Blue anti-mouse/rat/human CD27 antibody	BioLegend	124218
Pacific Blue anti-mouse/rat/human CD27 antibody	BioLegend	124217
PerCP/Cyanine5.5 anti-mouse CD223 (LAG-3) antibody	BioLegend	125212
PE/Cyanine7 anti-mouse CD279 (PD-1) antibody	BioLegend	109110
APC anti-mouse CD25 antibody	BioLegend	102012
APC anti-mouse CD62L antibody	BioLegend	104412
APC anti-mouse I-A/I-E antibody	BioLegend	107613
APC anti-mouse CD366 (Tim-3) antibody	BioLegend	134008
CD107a (LAMP-1) monoclonal antibody [eBio1D4B (1D4B)], eFluor 660	eBioscience	51-1071-82
FITC anti-mouse CD3 antibody	BioLegend	100204
PE/Dazzle 594 anti-human CD62L antibody	BioLegend	556419
FITC anti-mouse/human KLRG1 (MAFA) antibody	BioLegend	138410
FITC anti-mouse CD86 antibody	BioLegend	105110
PerCP/Cyanine5.5 anti-T-bet antibody	BioLegend	644806
FOXP3 monoclonal antibody (FJK-16s), eFluor 450	eBioscience	48-5773-82
PE anti-TCF1 (TCF7) antibody	BioLegend	564217
PerCP/Cyanine5.5 anti-mouse IFN-γ antibody	BioLegend	505822
Alexa Fluor 700 anti-human/mouse granzyme B recombinant antibody	BioLegend	372222
PE/Cyanine7 anti-mouse TNFα antibody	BioLegend	506324
PE anti-mouse IL-2 antibody	BioLegend	503808
APC anti-human IL-10 antibody	BioLegend	506807
PE anti-mouse perforin antibody	BioLegend	154406
PE anti-human CD8 antibody	BioLegend	344706
Recombinant Alexa Fluor 647 anti-GM130 antibody [EP892Y]	Abcam	ab195303
FITC anti-human CD3 antibody	BioLegend	317306
FITC anti-mouse CD279 (PD-1) antibody	BioLegend	135214
APC anti-human CD19 antibody	BioLegend	302212
PE/Cyanine7 anti-human CD8 antibody	BioLegend	344750
PE/Cyanine7 anti-human CD34 antibody	BioLegend	343516
PE/Cyanine7 anti-human CD279 (PD-1) antibody	BioLegend	329918
PE/Cyanine7 anti-human CD62L antibody	BioLegend	304822
APC/Cyanine7 anti-human CD8 antibody	BioLegend	344714
APC/Cyanine7 anti-human CD366 (Tim-3) antibody	BioLegend	345026
Brilliant Violet 711 anti-human CD223 (LAG-3) antibody	BioLegend	369319
PRDX4 Rabbit PolyAb	Proteintech	10703-1-AP
Anti-Giantin mouse	Abcam	ab37266
Anti-Giantin rabbit	Abcam	ab37266
Anti-Tom20 rabbit	Santa Cruz Biotechnology	sc-17764
Phospho-S6 ribosomal protein (Ser^235/236^)—Alexa647	Cell Signaling Technology	4851
Phospho-PERK (Thr^980^)	Cell Signaling Technology	3179
Anti-rabbit PE	Jackson ImmunoResearch Laboratories	111-116-144
Anti-rabbit Alexa 647	Abcam	Ab150079
Anti-rabbit Alexa 488	Abcam	Ab150077
Fetal bovine serum	Atlanta Biologicals	S11150
		
Bacterial and virus strains		
CBS (NM_000071) human tagged ORF clone	Origene	RC201755
		
Biological samples		
Healthy human PBMCs	Research Blood Components LLC	
		
Chemicals, peptides, and recombinant proteins		
GYY4137	Cayman	13345
NaHS	MilliporeSigma	161527
H_2_O_2_	Sigma-Aldrich	H1009
Gp100	GenScript	RP20344
Recombinant IL-2	NCI, Biological Resources Branch	https://ncifrederick.cancer.gov/research/brb/productDataSheets/cytokineHumanInterleukins/IL-2Bulk.aspx
Recombinant IL-4	R&D Systems	404-ML-025
Recombinant IL-6	BioLegend	575702
Recombinant IL-10	BioLegend	575802
Recombinant IL-12	BioLegend	573004
Recombinant IL-15	PeproTech	200-15
Recombinant TGFb	BioLegend	580702
Recombinant GM-CSF	BioLegend	576304
Monensin	BioLegend	420701
2-Deoxy-d-glucose (2DG)	Sigma-Aldrich	D6134
Antimycin A	Sigma-Aldrich	A8674
Rotenone	Sigma-Aldrich	R8875
Oligomycin	Sigma-Aldrich	O4876
FCCP	Sigma-Aldrich	C2920
IMDM	GE Healthcare, HyClone	SH30228.01
PST3.1a (NSC-753859)	Aobious	AOB13789
		
Critical commercial assays		
TET Hydroxylase Activity Quantification Kit (Fluorometric)	Abcam	ab156913
WSP-1	Cayman	11179
CellTrace Violet Cell Proliferation Kit	Thermo Fisher Scientific	C34557
Click-iT Plus OPP Alexa Fluor 647 Protein Synthesis Assay Kit	Thermo Fisher Scientific	C10458
FITC annexin V Apoptosis Detection Kit I	BD Pharmingen	556547
7-AAD	Thermo Fisher Scientific	A1310
MitoTracker Deep Red FM	Thermo Fisher Scientific	M22426
Tetramethylrhodamine, methyl ester, perchlorate (TMRM)	Thermo Fisher Scientific	T668
MitoSOX mitochondrial superoxide indicators	Thermo Fisher Scientific	M36008
ThiolTracker Violet (glutathione detection reagent)	Thermo Fisher Scientific	T10095
Alexa Fluor 633 C5 maleimide	Thermo Fisher Scientific	A20342
BODIPY FL C5-ceramide (*N*-(4,4-difluoro-5,7-dimethyl-4-bora-3a,4a-diaza-s-indacene-3-pentanoyl)sphingosine)	Thermo Fisher Scientific	D3521
H2DCFDA	Thermo Fisher Scientific	D399
NaveniFlex Cell MR RED	Cayman Chemical	39505
iScript cDNA Synthesis Kit	Bio-Rad	1708891
SsoAdvanced Universal SYBR Green Supermix	Bio-Rad	1725274
NE-PER nuclear and cytoplasmic extraction reagents	Thermo Fisher Scientific	78833
Nucleofector kits for mouse T cells	Lonza	VPA-1006
Fixation/Permeabilization Solution Kit	BD Biosciences	554714
Foxp3/Transcription Factor Staining Buffer Set	Thermo Fisher Scientific	00-5523
LIVE/DEAD Fixable Yellow Dead Cell Stain Kit	Thermo Fisher Scientific	L34959
RT^2^ profiler PCR arrays	Qiagen	PARN-034Z, PAZF-014Z
Tumor dissociation kit, mouse	Miltenyi	130-096-730
		
Deposited data		
Raw and analyzed RNA-seq data	This paper	GEO: GSE249287
Raw and analyzed proteomics data	This paper	PRIDE: PXD048141
		
Experimental models: Cell lines		
Jurkat	ATCC	TIB-152
Raji	ATCC	CCL-86
B16-F10	ATCC	CRL-6475
		
Experimental models: Organisms/strains		
Mouse: C57BL/6		000664
Mouse: B6-Rag−/−		034159
Mouse: Pmel		005023
Mouse: NSG		005557
Mouse: Cbs−/−		002461
		
Oligonucleotides		
Prdx4 siRNA	Ambion	AM16706
Primer: CBS, forward: GATCGCCAGAAAGCTGAAGGAG, reverse: CCACCTCATAGGCTGTTTGCTC	Invitrogen	A15612T
Primer: CSE, forward: GTGGGACAAGAGCCTGAGCAAT, reverse: GGATTTCCAGAGCGGCTGTATTC	Invitrogen	A15612T
Primer: GCLM, forward: CCTCGCCTCCGATTGAAGATG, reverse: AAAGGCAGTCAAATCTGGTGG	Invitrogen	A15612T
Primer: GCLC, forward: GGACAAACCCCAACCATCC, reverse: GTTGAACTCAGACATCGTTCCT	Invitrogen	A15612T
Prdx4 shRNA: pLV[shRNA]-Puro-U6>hPRDX4[shRNA#1]	VectorBuilder	MaxH(VB230622-1500vrc)
Prdx4 parent plasmid: pLV[Exp]-Neo-CMV>EGFP/hPRDX4[NM_006406.2]	VectorBuilder	MaxH(VB230622-1499hxg)
Prdx4 mutant primer: forward: CGGACTCGCGAAGAGGAGGCCCACTTCTAC, reverse: CCACCCGCGTAGAAGTGGGCCTCCTCTTC	Invitrogen	A15612T
Primer: MGAT1, forward: CCTATGACCGAGATTTCCTCGC, reverse: TGAAGCTGTCCCTGCCCGTATA	Invitrogen	A15612T
Primer MGAT5A, forward: AGGCAGAACCAGTCCCTTGTGT, reverse: CTTTGTGCTGGAGCCATAAACAG	Invitrogen	A15612T
Primer MGAT5B, forward: CTCTTACCGCAGCCTGAGTTTC, reverse: GCAGGAAGATGCAACCATTGGC	Invitrogen	A15612T
		
Software and algorithms		
FlowJo 10.2	TreeStar, OR	https://flowjo.com/solutions/flowjo/downloads/
Prism 8	GraphPad	https://graphpad.com/scientific-software/prism/
Agilent Seahorse Wave 2.4	Agilent	http://agilent.com/en-us/products/cell-analysis-(seahorse)/seahorse-wave-software
CFX Manager 3.1	Bio-Rad	https://bio-rad-cfx-manager.software.informer.com/3.1/
MaxQuant (MQ) v. 2.0.1.0	Max Planck Institute	PRIDE PXD048141
Perseus v. 1.6.15.0	Max Planck Institute	PRIDE PXD048141
