# Supplementary material for: H2S-Prdx4 axis mitigates Golgi stress to bolster tumor-reactive T cell immunotherapeutic response
Source: Sci Adv. 2024 Nov 15;10(46):eadp1152. doi: 10.1126/sciadv.adp1152 (PMC11566994; doi:10.1126/sciadv.adp1152)
Supplement: Supplementary file 1 — Figs. S1 to S7 Legends for tables S1 and S2 [file sciadv.adp1152_sm.pdf]

Science Advances
AAAS

# Supplementary Materials for

## $H_2S$-Prdx4 axis mitigates Golgi stress to bolster tumor-reactive T cell immunotherapeutic response

Nathaniel Oberholtzer *et al.*

Corresponding author: Shikhar Mehrotra, mehrotr@musc.edu

*Sci. Adv.* **10**, eadp1152 (2024)
DOI: 10.1126/sciadv.adp1152

**The PDF file includes:**

Figs. S1 to S7
Legends for tables S1 and S2

**Other Supplementary Material for this manuscript includes the following:**

Tables S1 and S2

**Supplementary Figure 1**

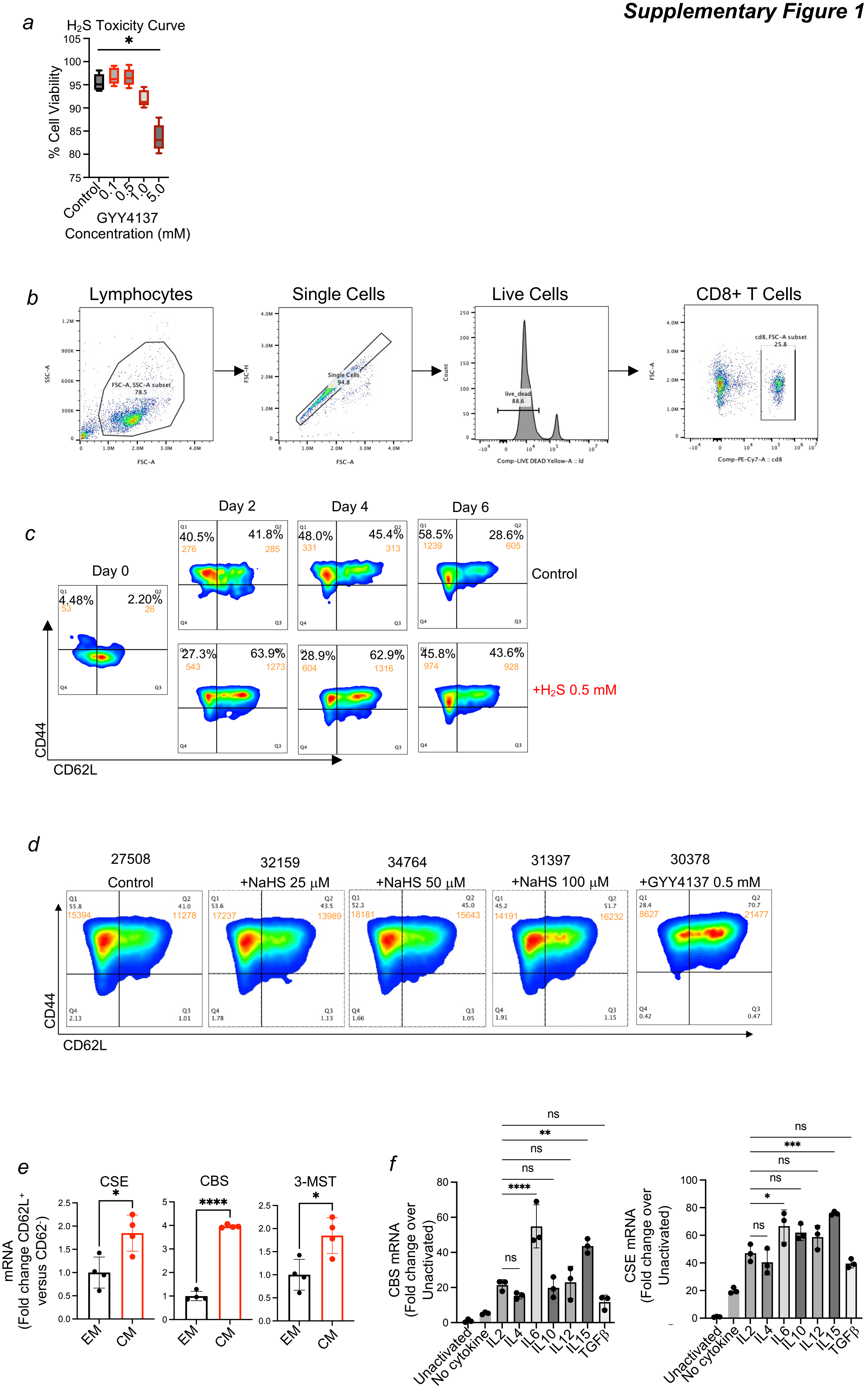

## ***Supplementary Figure 1***

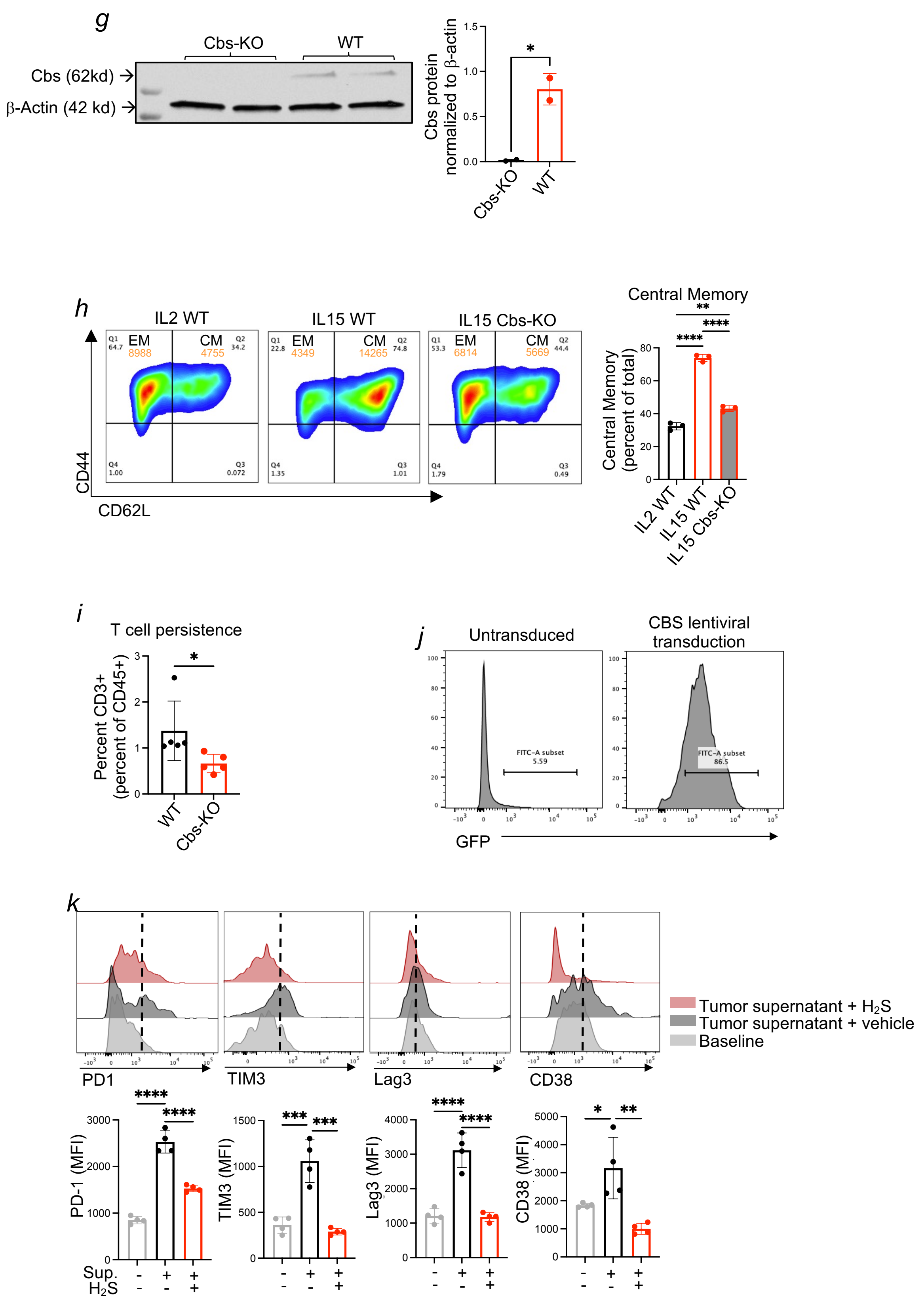

***Supplementary Figure 1. a***, Pmel T cells were stained with trypan blue stain on day 3 following activation with gp100 + IL2 (100 IU/ml) and treated with a range of concentrations of GYY4137 (n=5 independent samples). ***b***, General gating strategy for FACS analysis. Splenocytes isolated from the spleens of Pmel mice stained with LIVE/DEAD Fixable Yellow dye and anti-CD8 PE-Cy7. All FACS samples were first gated on the lymphocyte population based on FSA vs SSA. Single cells were gated using FSA vs. FSH. Live cells were gated using live/dead exclusion dye. To analyze Pmel T cells, CD8+ cells were gated by selecting the positive population. ***c,*** Pmel T cells were stimulated with gp100 tumor antigen and treated with either vehicle control or GYY4137 (0.5 mM) and analyzed by FACS for expression of CD62L and CD44 at day 0 (before stimulation), day 2, day 4, and day 6. ***d***, Pmel T cells activated in the presence of various concentrations of NaHS and analyzed using FACS for expression of CD62L and CD44. ***e,*** Pmel T cells were activated with gp100 for 3 days and were subsequently sorted by FACS based on expression of CD62L and CD44 to obtain effector memory (EM, $CD62L^-CD44^+$) and central memory (EM, $CD62L^+CD44^+$) populations. mRNA was isolated from each sorted population and RT-PCR was performed to determine relative mRNA levels of $H_2S$ producing enzymes (CSE, CBS, and 3-MST) (n=4). ***f,*** $CD8^+$ splenic T cells from gp100 TCR transgenic Pmel mouse were activated for 3 days in the presence of various cytokines and analyzed for mRNA expression of CBS and CSE. ***g,*** Splenocytes were isolated from wildtype and Cbs-knockout mice. Splenocytes were activated with anti-CD3 and anti-CD28 antibodies an analyzed for expression of Cbs by western blot. ***h,*** Splenocytes from WT and Cbs-KO mice (lacking the Cbs enzyme) were activated with anti-CD3 and anti-CD28 antibodies and expanded with either IL-2 under standard culture conditions or expanded with IL-15 (10 ng/mL) with or without addition of $H_2S$ to the Cbs-KO and analyzed for expression of CD62L and CD44 by FACS (n=3). ***i,*** T cells obtained from WT and Cbs-KO spleens were activated and expanded prior to adoptive transfer into $Rag^{-/-}$ mice ($1x10^6$ cells per mouse). At day 14, the mice were bled and the frequency of CD3+ T cells was assessed by FACS (n=5). ***j,*** Pmel T cells were transduced with either control or CBS lentivirus and transduction efficiency

was analyzed by assessing GFP expression by FACS. ***k*****,** Cells were analyzed using FACS for expression of PD1, TIM3, Lag3, and CD38 (n=3 independent samples). MFI represents mean fluorescence intensity. All data shown represent the mean ± SEM and were analyzed by two-sided Student's *t*-test or one-way ANOVA. ns, *p* value > 0.05; *p* value *≤ 0.05; ** ≤ 0.01; ***≤ 0.001; ****≤ 0.0001.

***Supplementary Figure 2***

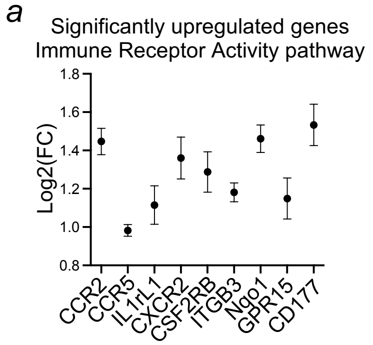


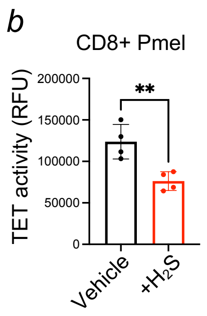

***Supplementary Figure 2.*** ***a***, Significantly upregulated genes identified in RNA-seq analysis. ***b***, Melanoma epitope gp100 TCR reactive CD8$^+$ T cells were activated for 3 days with gp100 and IL2 +/- 0.5mM GYY4137. On day 3, the cells were collected and TET activity was assessed using nuclear protein extract from CD8+ T cells treated with or without 0.5 mM GYY4137. Data shown represent the mean ± SEM and were analyzed by two-sided Student's *t*-test or one-way ANOVA, unless otherwise specified. ns, *p* value > 0.05; *p* value *≤ 0.05; ** ≤ 0.01; ***≤ 0.001; ****≤ 0.0001.

*Supplementary Figure 3*

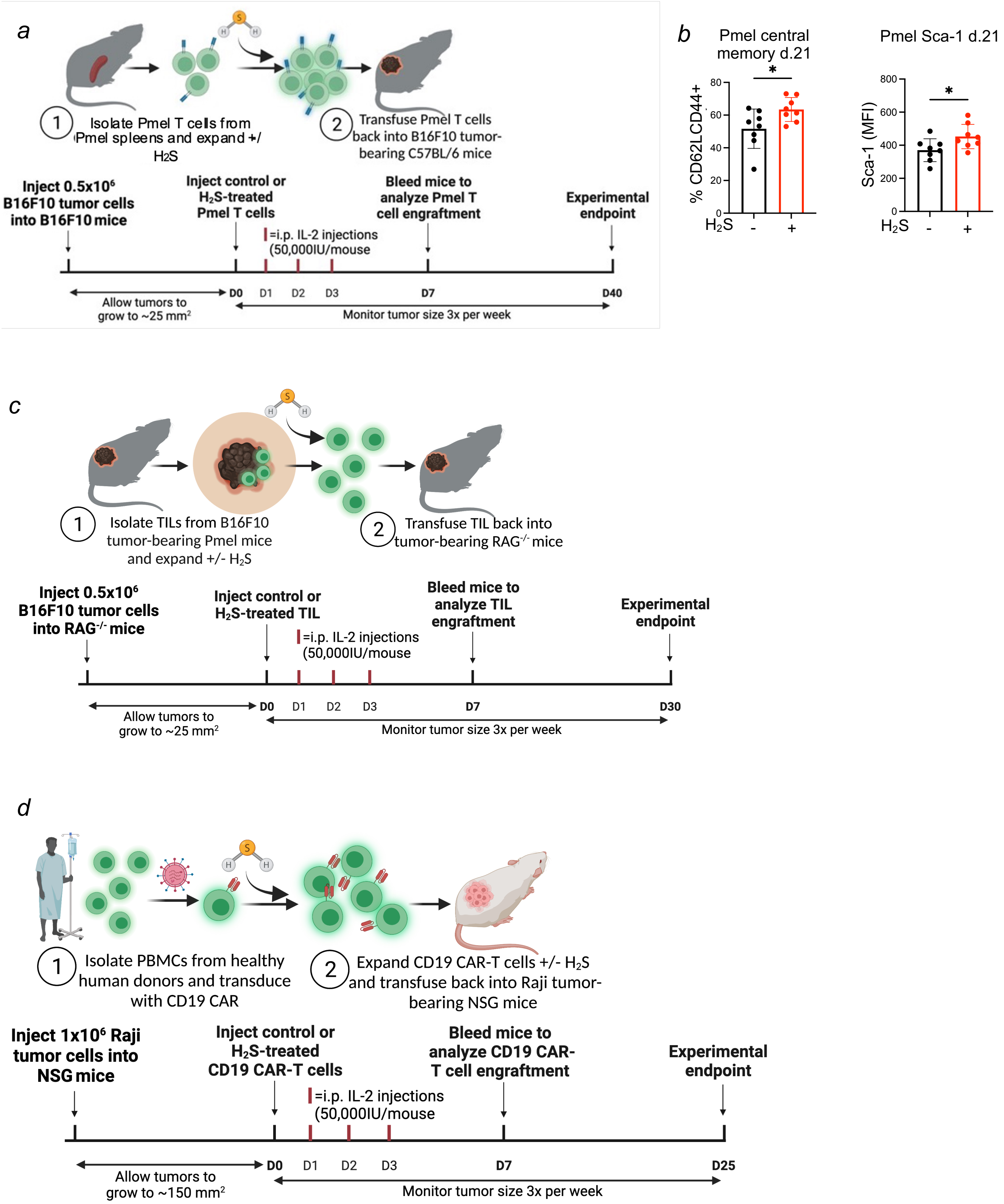

***Supplementary Figure 3***: ***a-d***, General schema for experiments using ***a,*** Pmel T cells with B16F10 melanoma tumors, ***c,*** TILs isolated from B16F10 melanoma tumors and used to treat B16F10 tumors in Rag$^{-/-}$ mice, or ***d,*** generation of human CD19-CAR-T cells to treat huma Raji lymphoma tumors in NSG mice. ***b,*** At day 21 following transfer of Pmel T cells into mice bearing B16F10 melanoma tumors, mice were bled and the retrieved cells were analyzed for expression of memory and stemness markers by FACS. All data shown represent the mean ± SEM and were analyzed by two-sided Student's *t*-test or one-way ANOVA. ns, *p* value > 0.05; *p* value *≤ 0.05; ** ≤ 0.01; ***≤ 0.001; ****≤ 0.0001.

*a*

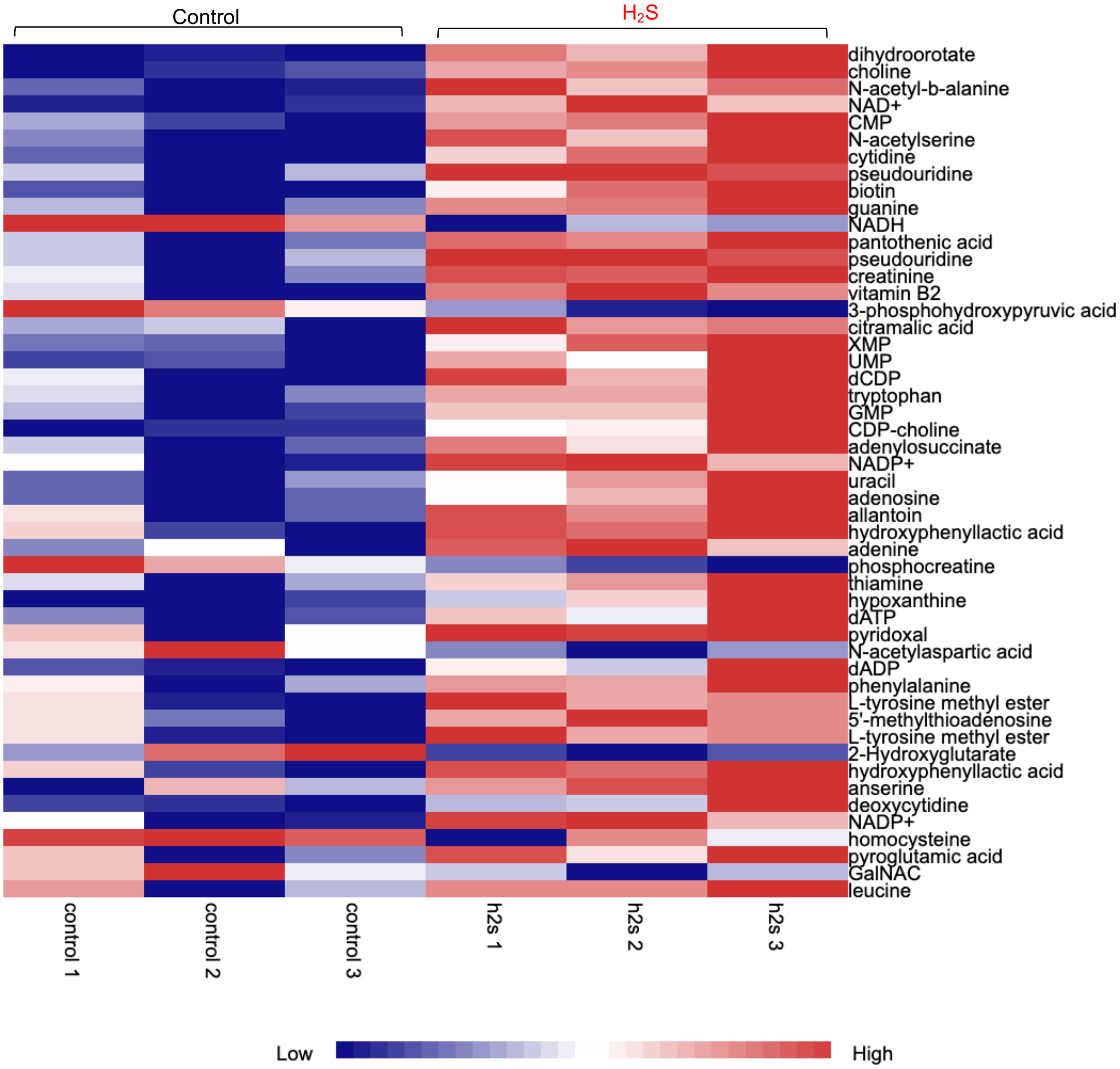

Control
$H_2S$
dihydroorotate
choline
N-acetyl-b-alanine
NAD+
CMP
N-acetylserine
cytidine
pseudouridine
biotin
guanine
NADH
pantothenic acid
pseudouridine
creatinine
vitamin B2
3-phosphohydroxypyruvic acid
citramalic acid
XMP
UMP
dCDP
tryptophan
GMP
CDP-choline
adenylosuccinate
NADP+
uracil
adenosine
allantoin
hydroxyphenyllactic acid
adenine
phosphocreatine
thiamine
hypoxanthine
dATP
pyridoxal
N-acetylaspartic acid
dADP
phenylalanine
L-tyrosine methyl ester
5'-methylthioadenosine
L-tyrosine methyl ester
2-Hydroxyglutarate
hydroxyphenyllactic acid
anserine
deoxycytidine
NADP+
homocysteine
pyroglutamic acid
GalNAC
leucine
control 1
control 2
control 3
h2s 1
h2s 2
h2s 3
Low
High

***Supplementary Figure 4*. *a***, significantly upregulated and downregulated metabolites identified in metabolomics screen comparing vehicle versus $H_2S$-treated T cells.

***Supplementary Figure 5***

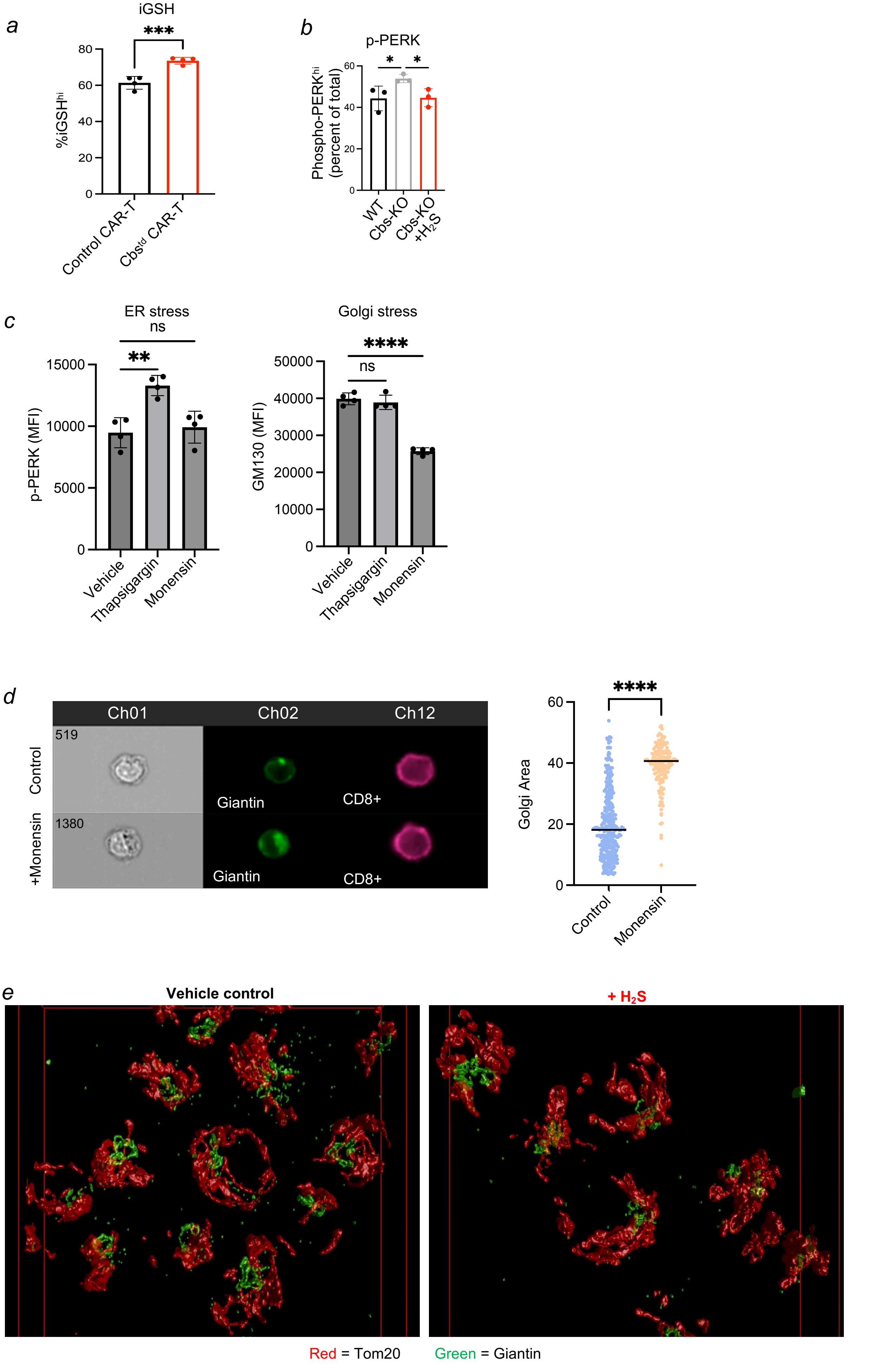

***Supplementary Figure 5. a***, Levels of intracellular glutathione assessed using FACS by employing ThiolTracker Violet dye in control CD19 CAR-T cells or CAR-T cells overexpressing Cbs ($Cbs^{td}$CD19-CAR-T) (n=3). ***b,*** 12 hour Thapsigargin (700 nM) treatment was used to induce ER stress in WT T cells, Cbs-KO T cells, and Cbs-KO T cells + $H_2S$. Levels of phosphorylated PERK were assessed by FACS (n=3). ***c***, Pmel T cells were treated with either Thapsigargin (700 nM) or Monensin (1 mM) for 12 hours, followed by staining for phosphorylated PERK (p-PERK) and GM130 to assess ER stress and Golgi stress, respectively (n=4). ***d***, Activated Pmel T cells were treated for 12 hours with either vehicle control or 1mM Monensin ("+Golgi stress") before being stained for giantin to assess Golgi dispersion. Image shows cells stained with anti-Giantin antibody and analyzed for Golgi dispersion (Golgi area) using ImageStream. ***e***, Representative confocal microscopy images of mitochondria (Tom20) and Golgi (giantin) in human PBMCs treated with vehicle control or 0.5 mM GYY4137. All data shown represent the mean ± SEM and were analyzed by two-sided Student's *t*-test or one-way ANOVA unless otherwise specified. ns, *p* value > 0.05; *p* value *≤ 0.05; ** ≤ 0.01; ***≤ 0.001; ****≤ 0.0001.

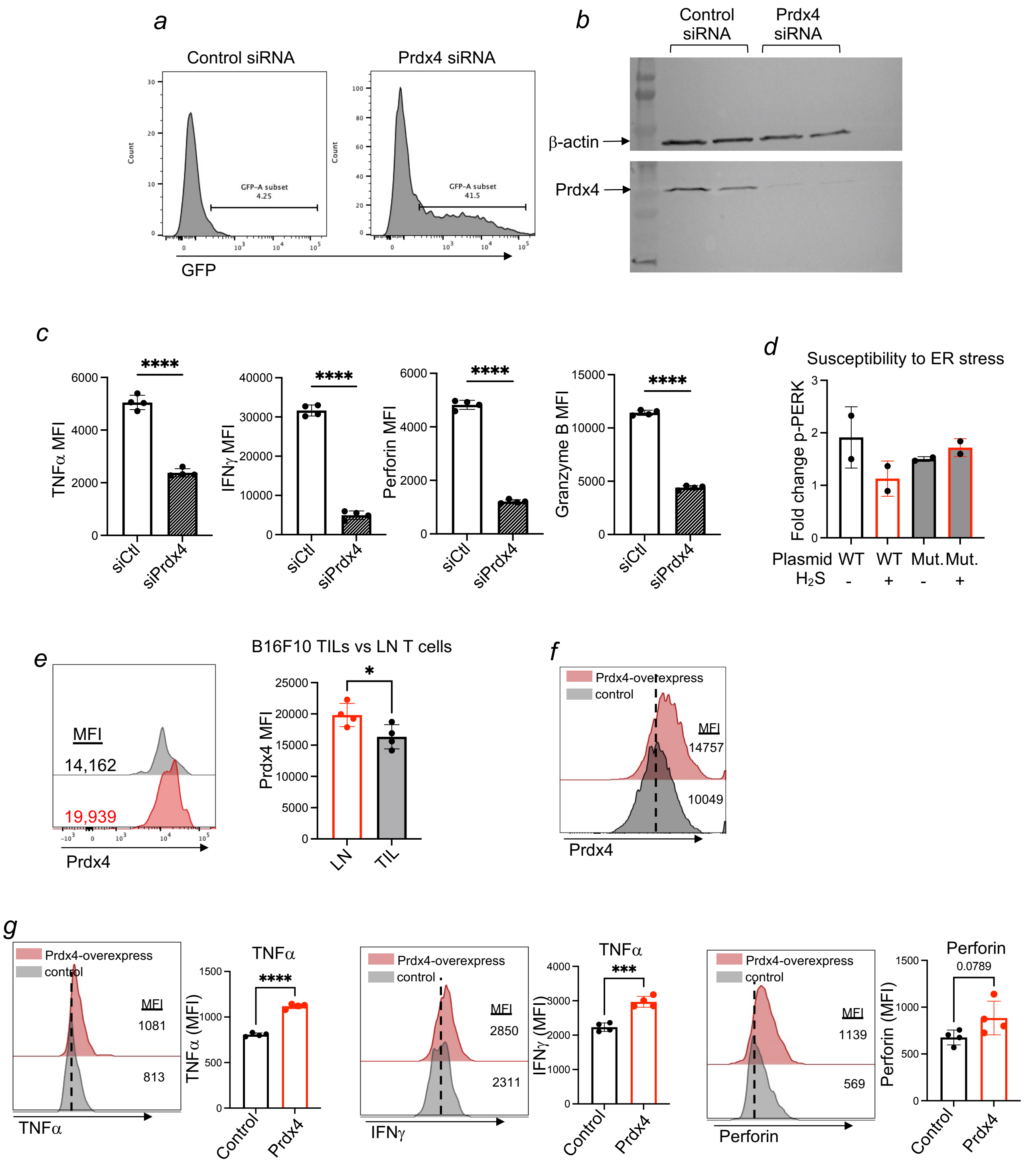
a
Control siRNA
Prdx4 siRNA
GFP-A subset
4.25
GFP-A subset
41.5
Count
GFP
b
Control siRNA
Prdx4 siRNA
β-actin
Prdx4
c
****
TNFα MFI
IFNγ MFI
Perforin MFI
Granzyme B MFI
siCtl
siPrdx4
d
Susceptibility to ER stress
Fold change p-PERK
Plasmid WT WT Mut. Mut.
H2S - + - +
e
MFI
14,162
19,939
Prdx4
B16F10 TILs vs LN T cells
*
Prdx4 MFI
LN
TIL
f
Prdx4-overexpress
control
MFI
14757
10049
Prdx4
g
Prdx4-overexpress
control
MFI
1081
813
TNFα
TNFα (MFI)
Control
Prdx4
2850
2311
IFNγ
***
IFNγ (MFI)
1139
569
Perforin
0.0789
Perforin (MFI)

***Supplementary Figure 6. a***, Transfection efficiency of control siRNA (GFP-) and siRNA directed against Prdx4 (GFP+) assessed via expression of GFP. ***b***, Expression of Prdx4 assessed by western blot. ***c***, FACS analysis of cytokine production by Pmel T cells transfected with either control siRNA (siCtl) or siRNA directed against Prdx4 (siPrdx4) (n=4). ***d***, Expression of phosphorylated-PERK in Prdx4-knockout Jurkat cells transfected with either wildtype Prdx4 plasmid or mutant Prdx4 plasmid (n=2). ***e,*** B16-F10 tumors were injected s.c. into the flanks of Pmel mice. Upon reaching tumor volume of approximately 100mm$^2$, tumor infiltrating lymphocytes (TILs) and T cells from tumor draining lymph nodes (LN) were isolated via Ficoll gradient spin followed by magnetic bead positive selection. FACS analysis of Prdx4 expression in PD1$^+$ TILs vs LN T cells (n=4). ***f-g***, Lentiviral supernatant was generated using either GFP-tagged control plasmid or GFP-tagged Prdx4-overexpression plasmid. Activated Pmel T cells were transduced with either control or Prdx4 lentivirus for subsequent assays. ***f,*** Overexpression of Prdx4 was confirmed by FACS. ***g***, Pmel T cells overexpressing Prdx4 and control T cells were restimulated with gp100 and analyzed for cytolytic cytokine production by FACS. All data shown represent the mean ± SEM and were analyzed by two-sided Student's *t*-test or one-way ANOVA unless otherwise specified. ns, *p* value > 0.05; *p* value *≤ 0.05; ** ≤ 0.01; ***≤ 0.001; ****≤ 0.0001.

# Supplementary Figure 7

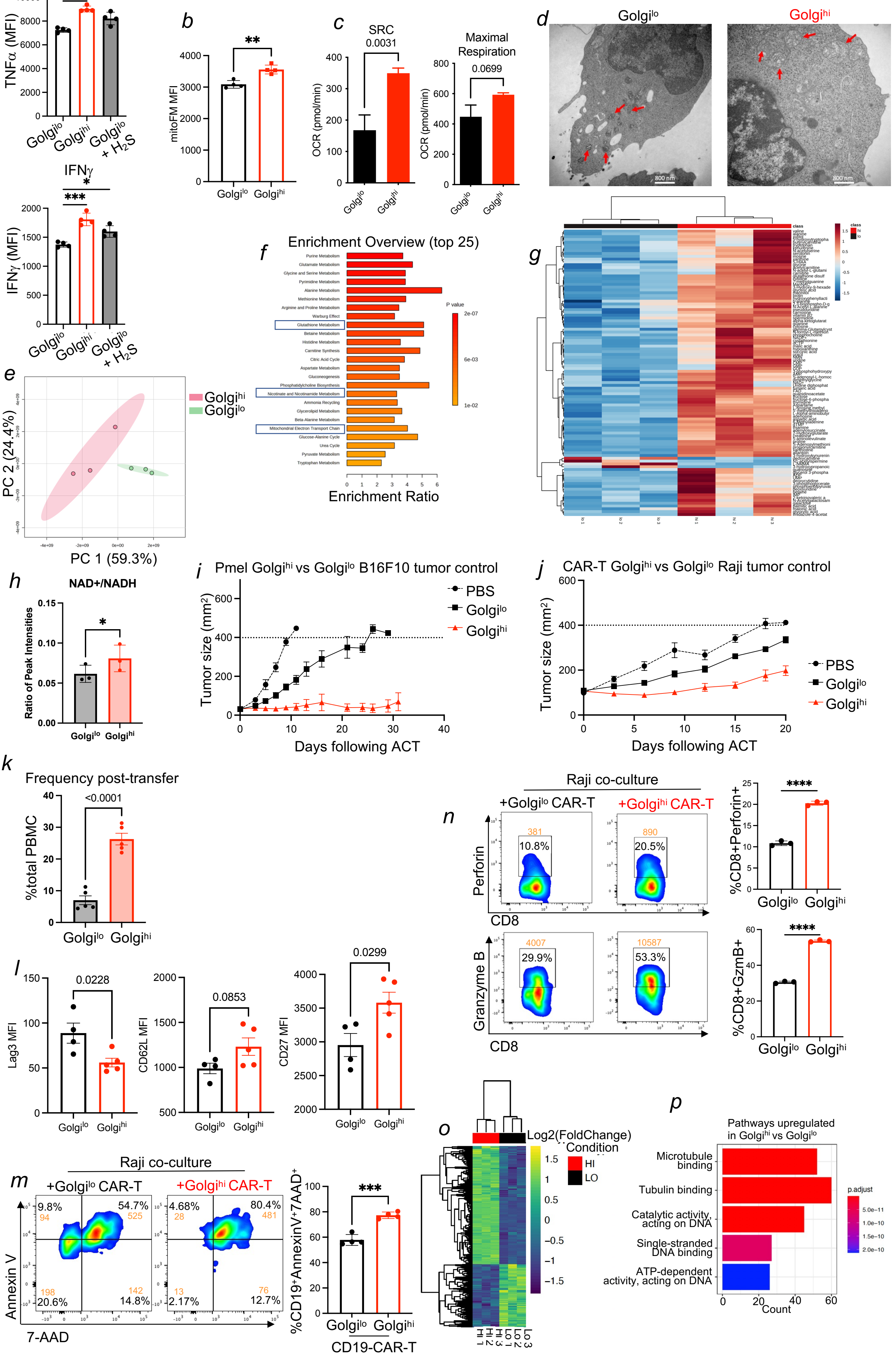

***Supplementary Figure 7***. ***a***, Activated Pmel T cells were sorted based on Golgi$^{hi}$ vs Golgi$^{lo}$ and expanded in culture with either vehicle control or GYY4137 (0.5 mM) for 3 days. Cells were subsequently stimulated with gp100 antigen and analyzed for production of cytokines by FACS (n=4). ***b***, Quantification of FACS MFI values for MitoTracker Deep Red FM stain in Golgi$^{hi}$ *vs.* Golgi$^{lo}$ cells (n= 4 independent samples). ***c***, Real-time flux in OCR measured using Seahorse metabolic flux analysis. Shown is the quantification of mean oxygen consumption rate (OCR) throughout the assay, spare respiratory capacity (SRC), maximal respiration, and basal respiration (n= experimental replicates representative of 3 separate experiments). ***d***, FACS sorted Golgi$^{hi}$ *vs.* Golgi$^{lo}$ melanoma epitope gp100 TCR reactive CD8$^{+}$ T cells from Pmel mouse were analyzed using transmission electron microscopy (TEM) to evaluate mitochondria and golgi morphology. ***e-h***, Global metabolomics analysis was performed on Golgi$^{hi}$ *vs.* Golgi$^{lo}$ Pmel T cells and analyzed for PCA (***e***) and pathway enrichment (***f***). ***g***, Significantly upregulated and downregulated metabolites (Golgi$^{hi}$ vs. Golgi$^{lo}$). ***h***, levels of NAD$^{+}$ and NADH obtained from metabolomics. ***i***, Tumor size measurements for C57BL/6 mice bearing B16-F10 tumors treated with FACS sorted T cells (n=10 mice per group). ***k***, At day 14 after transfer, the tumor-bearing mice were bled to analyze the frequency of circulating Pmel T cells and ***l***, for expression of surface markers using FACS. ***j,*** Tumor size measurements for Raji tumor-bearing NSG mice treated with FACS sorted CD19 CAR-T cells (n=10 mice per group). ***n-m,*** Human CD19 CAR-T cells were expanded and sorted into Golgihi and Golgilo based on BODIPY TR Ceramide dye fluorescence and were co-cultured at a 1:1 ratio with Raji cells. ***m,*** The percentage of viable Raji cells remaining after 24 hours was analyzed using FACS. ***n***, Expression of Perforin and Granzyme B was assessed using FACS in sorted CD19 CAR-T cells following co-culture with Raji cells. ***o-p***, bulk RNA-seq analysis performed on human CD19 CAR-T cells FACS sorted on Golgi$^{hi}$ vs Golgi$^{lo}$ (n=3 independent samples from 3 separate donors). ***o,*** heat map cluster analysis showing significantly up- and down-regulated genes. ***p***, significantly upregulated pathways in the Golgi$^{hi}$ subset identified by Gene Ontology (GO) enrichment analysis. All data shown represent the mean ± SEM

and were analyzed by two-sided Student's *t*-test or one-way ANOVA unless otherwise specified

ns, *p* value > 0.05; *p* value *≤ 0.05; ** ≤ 0.01; ***≤ 0.001; ****≤ 0.0001.

***Supplementary Table 1*.** Proteomics data probing for cysteine containing peptides with thiols sensitive to $H_2S$. Out of 6,991 cysteine containing peptides identified, 770 were quantified with stable isotope labeled heavy (treated with $H_2S$) and light (control) iodoacetamide in all 3 biological replicate experiments.

***Supplementary Table 2*.** Label free proteomics data probing for proteins changing in relative abundance in response to $H_2S$ treatment. Out of 3,172 proteins identified, 2,219 were quantified in 100% of samples in the treated or control group.
